# Influence of Granulometry on Thermal and Mechanical Properties of Cement Mortars Containing Expanded Perlite as a Lightweight Aggregate

**DOI:** 10.3390/ma12234013

**Published:** 2019-12-03

**Authors:** Matías Leyton-Vergara, Alexis Pérez-Fargallo, Jesús Pulido-Arcas, Galo Cárdenas-Triviño, Jeremy Piggot-Navarrete

**Affiliations:** 1 Department of Building Science, University of Bio-Bio, Concepción 4030000, Chile; matias.leyton.v@gmail.com (M.L.-V.); aperezf@ubiobio.cl (A.P.-F.); jpulido@ubiobio.cl (J.P.-A.); 2 Department of Wood Engineering, University of Bio-Bio, Concepción 4030000, Chile; gcardenas@ubiobio.cl; 3 Department of Design and Theory of Architecture, University of Bio-Bio, Concepción 4030000, Chile

**Keywords:** thermal conductivity, fineness modulus, expanded perlite, mechanical strength, lightweight mortar

## Abstract

This research aims at clarifying the influence of the granulometry of expanded perlite, on the thermal conductivity, structural strength, density, and water absorption of lightweight mortars. Three original perlite gradations have been obtained and three pairs of twin test mortars have been tested with those gradations. SEM tests have also been run to clarify the interaction, at a microscopic level, between the expanded perlite and the cement grouting. The results indicate that the mere manipulation of the granulometry may have a considerable and very beneficial effect on the mixture’s properties, such as thermal conductivity and water absorption.

## 1. Introduction

Buildings are one of the world’s largest energy consumers, and it is estimated that they consume around 20%–40% of the energy in developed countries and around 24% in developing economies; however, the figures of the latter are expected to surpass the former between 2020–2025 [1]. In this context, adequate insulation of buildings remains crucial to contain this energy expenditure. Although there are differences between different classifications, in general, construction materials are classified as insulators if their thermal conductivity (λ) is below 0.1. The reduction of the thermal conductivity contributes directly to reducing the thermal bridges produced in the building’s envelope [2,3,4]. Among materials commonly used in construction, those with a cement base are especially important, with concrete and mortar included within these. As commonly used construction materials, their properties are regulated by domestic standards; in the case of Chile, where this study was conducted, local regulations provide a database for thermal conductivity including commercial products, like the case of non-structural mortar, where the λ value is set at 1.4 W/m·K for a density of 2000 kg/m^3^; for concrete, this value is set as 1.63 W/m·K for a density of 2400 kg/m^3^ (normal concrete) and 0.33 W/m·K for a density of 1000 kg/m^3^ (lightweight concrete).

Both mortar and concrete are cementitious materials that contain aggregates. Reducing the λ coefficient of these products and, thus, the heat losses in the building’s envelope, is one of the main goals of research related to mortar and concrete and it has been proved that this value is affected by the complex interplay of different variables, where lightweight aggregates play a crucial role.

Regarding mortars, recent studies have shown that a change in lightweight aggregates can reduce their thermal conductivity, while the structural compressive and flexural strength is also reduced [5]. There does not seem to be a simple explanation for this phenomenon, but according to background research, there are some factors that can be mentioned: the type of aggregate, its absorptivity, mineralogic composition, porosity, granulometry, and the characteristics of the particles. Different studies have been made to evaluate the influence of lightweight aggregates (pumice, diatomite, perlite, expanded perlite (EP), clay, among others) in the properties of the concrete [2,3,4,6,7,8,9,10,11,12,13,14]. All aggregates whose densities are less than 1120 kg/m^3^ are considered lightweight aggregates [7]. Their particular characteristics, like their porosity and the presence of interior air micro-cells, make them especially suitable to be added to mortars and concretes, since they reduce their thermal conductivity [8,9,15]. Higher moisture has also been found to be associated to higher λ coefficients for cement mortar, cement and lime mortar, and mortar modified with polypropylene fiber. For the 3 types, the conductivity ranges from 0.70 (W/m·K) to values close to 3 (W/m·K) [16]. 

However, most of the research on lightweight aggregates has been focused on concrete, which is a widely used construction material given its good acoustic and structural properties, even though its thermal properties are generally poor. That is why lightweight concrete (LC), containing coarse and fine lightweight aggregates, has been gaining momentum in recent times. LC, with its density below 1950 kg/m^3^, can be used in internal and external walls, inner leaves of external cavity walls, fill panels, insulation materials, among others [13]. There are many studies that deal with EP and diatomite, and they seem to produce the lowest thermal conductivities (between 0.6 W/m·K and 0.13 W/m·K) [9,12]. This research focuses specifically on EP, so a context is provided below regarding the state of the art for this material. 

EP has a foamy microstructure which provides excellent thermal insulation and high porosity properties to the low-density material that has been used in different studies [4,12,14,17,18,19,20,21,22,23]. The production of EP generates an important fine fraction percentage (5%–10%), with a particle size below 200 μm. Its only problem is its large specific surface area, which generates major water absorption [24]. The EP stands out, not just on being one of the lightest aggregates when compared to expanded clay, expanded glass, and exfoliated vermiculite [25], but also due to its porous microstructure which provides excellent thermal properties when added to mortars and concretes [4,10,23,24,26,27,28]. What is more, it is non-toxic, fire resistant, has good acoustic insulation properties, has a competitive price, and does not react or leach into ground water [4,22,27,29,30,31,32]. 

The following studies can be highlighted among those that research the influence of EP on the properties of mortars and concrete, although most of them are focused on the latter. The addition of EP with a particle size of between 2 and 4 mm instead of sand as a natural aggregate for concrete, considerably reduced the density of the final product (392 kg/m^3^) [4]. It also reduced its thermal conductivity by 78% (from 0.6 to 0.13 W/m·K). On the other hand, the structural strength was found to be negligible, and a negative correlation between water absorption and density was found. However, the EP’s granulometry as an independent variable was not considered in this study. It has also been proven that the addition of lightweight aggregates comprising an EP covered with cement, fly ash, and expanded perlite powder have an effect on the structural and thermal properties of the concrete [33]. 

Likewise, it seems necessary to find a balance between density, structural strength, and thermal conductivity, and it is here where the complex interplay between EP and other components, at a macro and microscopic level, comes into play. The study of Wang et al., 2018, defined an optimal gradation of EP particles filled with aerogel in structural LC based on the numerical tube pressure (KPa) of the mixtures as an indicator [34], but not in the thermal conductivity. Several studies express that EP (without aerogel) has high hydrophilic properties [26,30,35], as such it tends to considerably increase the water absorption of construction materials like concrete or mortar when added to them [22,34,36]. There are studies which take advantage of the hydrophilic properties of EP to increase the humidity contents in the concrete. Polat et al., 2015, incorporated different proportions of pre-saturated expanded perlite aggregate as an internal curing agent in concrete to reduce its autogenous shrinkage [21]. However, so that the EP positively affects the thermal properties of mortars and LC, it cannot be saturated as this may cause greater water requirements in the mixture and directly affect its proper functioning, since the proportion between liquids and solids in the mix directly affects the volume of the mortars and LC’s internal micro-cavities [22]. In addition, it must be considered that higher moisture content might imply higher heat transfer coefficients [37], as can be seen in the study made by Real et al., 2016 [2]. In fact, some studies have tried to limit the water absorption by the perlite by covering it with hexafluorobutyl acrylate (HFBA) [35] or filling it with aerogel [26,34], to also improve its thermal properties. 

Summing up, the state-of-the-art indicates that the interaction of EP with other concrete and mortar components is very complex, and because of that there have been many studies dedicated to clarifying, among other aspects, the relationship between the hygroscopic properties of the EP and the structural and hygrothermal properties of concrete and mortar. However, no relevant studies have been made about the influence of the EP’s granulometry on the hygrothermal and structural properties of mortars. The particle size is closely related to certain hygroscopic properties of the EP, water absorption above all. Therefore, this study is focused on clarifying whether the granulometry of the EP, used as a unique lightweight aggregate in a mortar, noticeably affects its structural and hygrothermal properties.

An experiment has been designed to clarify this question, where test mortars have been prepared, in which the water/cement (W/C) ratio is kept constant and EP has been exclusively used as a fine lightweight aggregate with different granulometries, characterized by its fineness modulus; an additive has not been used with the goal of solely analyzing the influence of the granulometry on the thermal conductivity, density, degree of water absorption, compressive, and flexural strength. These control conditions have been established to isolate the possible influence of the cement or additive content on the thermal conductivity and the structural strength of the mortar.

## 2. Materials and Methods 

### 2.1. Material Characterization

The EP, the main material in this study, was characterized by its elemental chemical composition, its apparent and compacted densities, and finally, its granulometry. Given that EP, as mentioned before, is a natural material subjected to an expansion process by adding heat, its elemental chemical composition was determined first, by an atomic absorption spectrometry analysis, obtaining the following chemical composition (Table 1).

Two types of densities where considered for the lightweight aggregate: loose apparent density and loose compacted density. Both were determined using the NCh 1116 Of. 2008 method [38], which is an adaptation of the ASTM C 29-71 standard [39]. Loose apparent density is defined as the quotient between the mass of the arid and its uncompacted volume; loose compacted density describes the mass of the arid and its volume, but after compacting three layers of the material of equal thickness with 25 ram strokes. Two twin samples were used for each type of EP (Table 2), so the resulting densities are the average for each pair of samples. The initial densities of the EP particles correspond to the commercial denominations PP6 and PP8, with the loose apparent density being 76 and 79 kg/cm^3^ and the compacted, 94 and 92 kg/cm^3^. The loose apparent densities for the modified granulometries fluctuated between 76 and 86 kg/cm^3^ and for the compacted between 99 and 111 kg/cm^3^, with the densities of the coarser granulometries being lower.

An original procedure was followed to determine the EP’s water absorption, since existing standards, like the NCh 1239 Of. 2019 [40], an adaptation of the ASTM C-128 15 standard [41], only include procedures to determine this parameter for aggregates that are denser than water (i.e., sand). For this reason, a mass difference water absorption test, which is normally used for clay, was run. In this case, 2 gr of EP was soaked in 100 mL of water at 14 °C for 2 h, while the mixture was being stirred using a magnetic stirrer. After that, the excess water was drained from the EP and the mass difference was measured with a precision of 0.01 gr. Water absorption values of 30% were obtained at 14 °C, which coincide with the data given by the supplier and with the values provided by similar studies [36].

The initial size distributions of the EP particles correspond to the commercial denominations PP6 and PP8. Both granulometries are over the grading requirements set out by the ASTM C332-17 standard for light aggregates used in concrete [42]. The cement used, was a II/B-P cement as per the EN 197-1 standard [43] and IP as per ASTM C 595 [44], with a specific weight of 2.8 gr/cm^3^ and a compressive strength at 28 days of 40.21 MPa. 

### 2.2. Mix Proportion and Mixtures 

**Figure 1:**
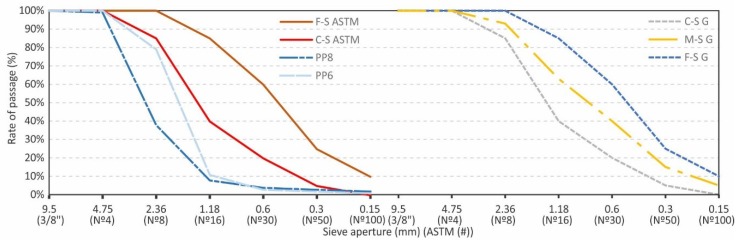
Expanded perlite grading PP6 and PP8 (left); grading groups (right).

In the experimental study, the first step consisted of creating three customized granulometries from the commercial PP6 and PP8 EP. In this way, one was generated for fine granulometry (F-SG), another for coarse granulometry (C-SG), and a third for medium granulometry (M-SG) (Figure 1) to analyze the influence of said parameter on the mixtures. The F-SG and M-SG had a maximum nominal particle size of 2.36 mm and the C-SG of 4.75 mm. The perlite was sieved to reach these three distributions with a set of standard calibrated sieves with apertures of 9.50 mm (3/8”), 4.75 mm (N°4), 2.36 mm (N°8), 1.18 mm (N°16), 0.60 mm (N°30), 0.30 mm (N°50), and 0.15 mm (N°100).

It is important to highlight that the granulometries prepared are based on the edge conditions of the ASTM C322-17 standard [42], where the coarse (C-S-ASTM) and fine (F-S-ASTM) coincide with the higher and lower limits; the mean granulometry is exactly the arithmetic mean between both (M-SG). 

The fineness modulus of the three granulometries was determined following the ASTM C 136 -14 [45] and ASTM C 332-17 [42] using minimum samples of 50 g for the three granulometries, giving results of 3.50 for C-SG, 2.85 for M-SG, and 2.20 for F-SG. The fineness module (FM) for each sample, following this procedure, represents the coarseness of a granulated material; a higher FM means coarser particles and lower FM means that the particles are smaller in size. The FM was determined as the sum of the accumulated percentages that were retained on each one of the following sieves: 4.75, 2.36, 1.18, 0.600, 0.300, and 0.150 mm; that quantity is divided by 100 and expressed as a percentage. The particle size distribution was determined with 203 mm diameter W.S. Tyler test sieves, that comply with ASTM E-11.

Previous research had proven that a reduction of the cement content and its substitution with other mineral products can reduce the conductivity to 0.792 W/m·K, but also reduce the compressive strength [46]. Little research has been done on a systematized method for dosing lightweight concrete, although those materials can find application in a wide range of contexts [47]. Because of this, of having a grounded guide about the appropriate amounts of cement, the proportion of the mixtures was based on previous research [48], where work was done with different amounts of cement in terms of the volumetric ratio with the perlite; the lowest amount of cement used is 175 kg/m^3^. In addition, according to the English dosing method for concrete that the Chilean NCh 170 Of. 2016 norm follows [49], the minimum amount of cement for uncontrolled concrete was set at 170 kg/m^3^. The mortar samples were then prepared with these three granulometric distributions. The proportions of the samples were made with 170 kg/m^3^ of cement and 81 kg/m^3^ of EP, with those values kept constant for all samples (Table 3). The water/cement ratio was 1.34 and was also kept constant; this value was determined from the results of pilot tests that determined that the cement dosage had to be increased to improve the workability of the mixture. The perlite was not subjected to pre-absorption so that the particle, in the result, was capable of absorbing the highest amount of cement grouting possible; that would allow observation under the microscope how this situation affected the material’s foamy microstructure; besides, it was not subjected to any grinding process. The use of the perlite without pre-absorption also explains the high relation between water and cement; not only the cement, but also the perlite was expected to absorb water, so the amount of water had to be increased. No additives or additions were used in the mixtures. In this way, it was possible to set the control conditions and isolate the granulometry of the perlite as the unique independent variable in the study.

Once the doses and the minimum cement dosage were determined, the test mortars were prepared for later testing, considering the results of Table 3. Fineness modulus ranged from 2.20 to 3.50, with the lowest value for the F-SG distribution and the highest for the C-SG (Table 3). All of them were prepared manually, always respecting the same order. First the EP and the arid were carefully mixed so that the aggregate’s granulometry was not altered; then water was added until it reached a homogeneous mixture. The mixture was poured into molds that were compacted manually and gently to reduce the air trapped in the mass of the mortar, since excessive compacting may cause segregation. 

All the samples were kept in their molds for 24 h, and after that were released from the molds and stored in a humid chamber with controlled conditions: temperature of 20 °C and 96% humidity for 28 days. After this, the samples were removed from the chamber and tested under laboratory conditions (50% of HR at approximately 20 °C).

### 2.3. Test Procedures

The following tests were conducted: thermal conductivity, water absorption, and compressive strength. Their dimensions and the weight (or mass) of the samples were also measured to determine the dry and humid densities.

The thermal conductivity was measured using a guard ring following the Chilean NCh 850.Of 2008 standard [50], which is a partial adaptation of the ASTM C177-19 standard [51]. The standardized and widely known procedure, comprises measuring the amount of heat transmitted by the material between its two opposing, flat, and parallel faces, inducing a constant temperature difference. Two twin samples made of the same material measuring 30 cm × 30 cm × 3 cm were used for each test. Likewise, the dry and humid density of the samples were measured before the thermal conductivity test. Mass was registered using a precision scale with an accuracy of 0.01 gr and the size of the samples was measured with a caliper, with a precision of 0.01 mm; in this way, the mass, volume and, therefore the density, can be determined. The humid density was measured immediately after the samples were taken from the humidity chamber after 28 days of curing, that is, in a humid state. After that, the samples were dried for 24 h in an oven at 60 °C and finally, their dry density was registered in a dry state. 

The thermal conductivity test was conducted after the dry humidity of the samples was registered. In total, the test lasted 24 h. The test mortars were then placed in the conductivity meter, which has a cold and a hot face that induce the temperature difference between the opposing faces of the samples. Six thermocouples were placed on each face (12 in total) and, once the system reached a stationary state, 18 measurements were taken, every 30 min, of the mean temperature of the 6 thermocouples on both faces (W/m^2^·K). The thermal conductivity (W/m^2^·K), along with the humidity of the material (%), the dry and humid density (kg/m^3^), and the moisture content (%) were determined in this process.

The water absorption was determined using the ASTM C 642-13 method [52]. The mass difference before and after submersion was used to calculate the water absorption percentage. The initial mass (dry mass) was determined with the test mortars dried at a constant mass in an oven at a temperature of 60 ± 5 °C; later, the test mortars were submerged in water at 21 °C for at least 48 h. The saturated mass was then measured, once extracted from the water. The test mortars were then left to dry naturally and then weighed again, until the mass difference between weighing at 24-hour intervals was less than 0.5% of the highest value.

The mechanical compressive strength was determined using the methodology of NCh 158 Of:1969 [53], which is a partial adaptation of the ASTM C109/C109M–16a standard [54]. Then, 40 mm × 40 mm × 40 mm test mortars were prepared, which were tested in a control uniframe press calibrated with a 10 kN load ring. The load surface was 1600 mm^2^. The load was applied uniformly and progressively, between 0.98 and 1.96 MPa/s, until the test mortar broke. Each test was performed with 3 twin test mortars of each dose and the results were the average of those 3 results.

The morphology and existence of elements in the samples were obtained using a scanning electron microscope (SEM), a Hitachi SU 3500 model (Hitachi, Ltd., Mexico City, Mexico) with an accelerating voltage of 10.0 kV. The SEM was coupled with a Bruker Quantax 100 energy dispersive X-ray spectroscope (EDX, Hitachi, Ltd.) for semi-quantitative determinations. The system had 7 nm secondary electrons imaging resolution at 3 kV accelerating voltage and 10 nm back scattered electrons imaging resolution at 5 kV accelerating voltage (High vacuum mode), an accelerating voltage range of 0.3–30 kV, and a variable pressure range of 6–650 Pa. Those tests were run to clarify how the porosity, size, and specific surface of the EP might have an influence on the interaction with the cement grouting. This interference is essential to understand how the analyzed properties are affected by the particle size due to the silting of the aggregate’s cavities by the cement. Because of this, the following images were produced: aggregates in natural state, aggregates in contact with the cement mortar, behavior of the cement grouting with the aggregate on the surface, and penetration in the aggregate. The mortar samples were ground for this, the particles were classified as per their granulometry, and the large ones were cut to see the changes in their internal structure.

## 3. Results

### 3.1. Density and Fineness Modulus

The fineness modulus, which represents the average size of the particles of a coarse aggregate by an index number, is used to express the gradation and the coarseness of the particles of an aggregate. The results for the EP show that, in general, the particle size is small and falls within industry standards. For example, the fineness modulus of sand used to manufacture standard concrete is between 1.6 and 3.7 [55]. The results of the granulometries are within this range, being between 2.20 and 3.50 (Table 3). On the other hand, the density in humid state obtained for the samples is approximately between 540 and 1010 kg/m^3^ and the density in dry state is between 376 and 660 kg/m^3^ (Table 4).

**Figure 2:**
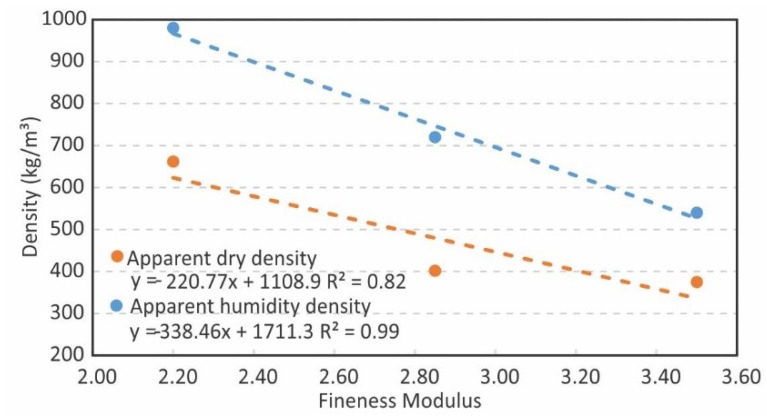
Effect of expanded perlite fineness modulus on the density.

The relationship between the fineness modulus and the density suggests that, the higher the fineness of the lightweight aggregate, the higher the density of the mortar, increasing, in both cases, by between 43.4% and 44.9% from the C-SG to F-SG samples. This was verified for both the apparent dry density and the apparent humidity density (Figure 2). For each density type, a linear relation can be established between these and the EP’s fineness modulus. It can also be seen that the mere act of manipulating the EP’s granulometry can have a considerable effect on the density of the resulting material. Likewise, it is seen that the relationship between the dry and humid density is not proportional, with the F-SG dose including a higher humidity content versus the mass, which initially indicates that the higher the FM, the lower the hygroscopy is.

### 3.2. Compressive Strength

**Figure 3:**
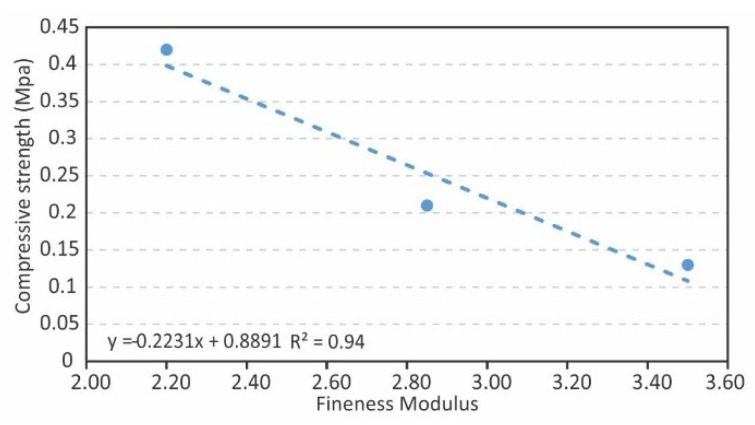
Effect of expanded perlite fineness modulus on the compressive strength.

A plausible correlation was found between the EP’s fineness module and the compressive strength of the samples (Figure 3); said relationship is linear with a correlation coefficient of 0.94. Considering these results together with those from Table 4, it can be suggested that there is a three-band relationship between the fineness modulus, density, and compressive strength. The higher the fineness modulus is, the lower the compressive strength is, and the lower the densities are, with the strength being reduced 69% between the F-SG to the C-SG. This indicates that the compactness of the small granulometries, as may be obvious, is greater than those prepared with coarser granulometries and, therefore, provides greater mechanical strength. 

In this sense, it seems interesting to suggest that, if the fineness modulus is taken as an independent variable, not only does the density depend on it (Figure 2), but compressive strength is also, simultaneously, a function of the others (Figure 3).

### 3.3. Absorption

**Figure 4:**
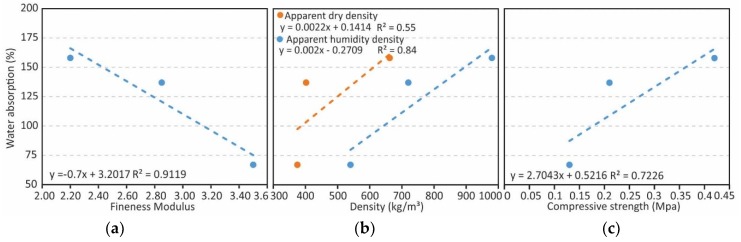
(**a**) Relation between water absorption and fineness modulus, (**b**) density, and (**c**) compressive strength.

The three pairs of twin samples have shown inconsistent values in water absorption, as such it appears logical to examine the relationship that this variable has with others (Figure 4). First of all, there seems to be a negative correlation with an R^2^ of 0.91 between the fineness modulus and the percentage of water absorption. The linear relationship suggests that coarse lightweight aggregates limit water absorption (Figure 4a). Therefore, it is confirmed that the hygroscopy is lower the higher the fineness modulus is, being able to drop 91%. This is due to the highly porous foamy microstructure that is characteristic of these aggregates [24], which form closed porosities if the aggregate has a suitable size.

The relation between the density of the samples and the water absorption percentage seems pretty weak, especially for the dry samples (R^2^ of 0.84 and 0.55). The humidity density of the samples and the absorption percentage has a closer relation given that both are related with the number of open pores in the material, as can be seen by the results of the F-SG sample, which has the highest number of open pores versus its apparent density. Finally, the correlation between the compressive strength and water absorption is linear, positive, and with a R^2^ coefficient of 0.72, leading to the thought that a higher water absorption percentage does not necessarily indicate a reduction of compressive strength, and confirming that a higher open porosity in this type of material implies a higher compressive strength.

### 3.4. Thermal Conductivuty

**Figure 5:**
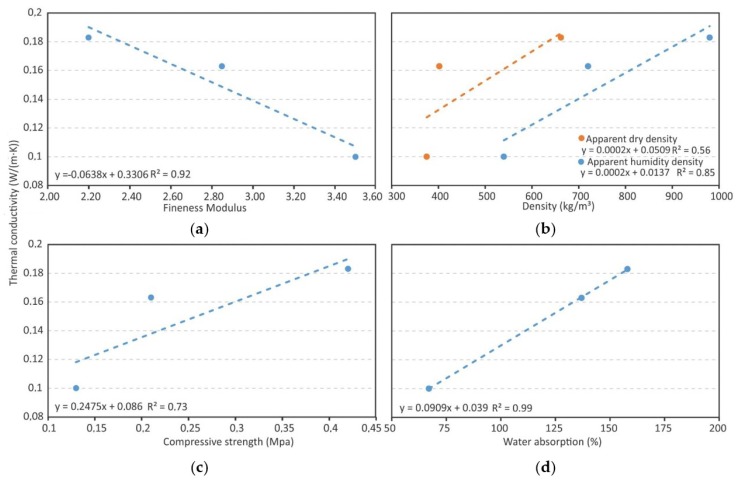
Relation between thermal conductivity and (**a**) fineness modulus, (**b**) density, (**c**) compressive strength, and (**d**) water absorption.

Certain relationships between thermal conductivity and other variables in the study could be clarified (Figure 5). First of all, considering the fineness modulus, the results suggest an evident correlation with thermal conductivity (Figure 5a). For coarse lightweight aggregates, the conductivity falls. Only by modifying the fineness modulus between 2.20 and 3.50, can the conductivity be reduced by 45%; therefore, choosing the maximum (C-SG) or minimum (F-SG) granulometry established by the ASTM C332-17 standard can provide very different conductivities [42]. As was mentioned in the introduction, there are two relationships which tend to be complied with for mortars: The higher the density, the higher the conductivity is, and the higher the compressive strength, the higher the thermal conductivity is. Said relations are confirmed in this study, although care must be taken as confidence levels are not at all satisfactory. This is especially true in the case of the dry density, where its correlation with conductivity is not clear. It is also noteworthy in that a 45% reduction of the thermal conductivity implies a 69% reduction in the compressive strength. The relation that appears to be more evident is the one between water absorption and conductivity, with pretty high confidence levels (99%), implying that a 91% reduction in the absorption represents a 45% reduction in the thermal conductivity.

### 3.5. SEM Analysis of Expanded Perlite and Cement Interface

The interaction between the lightweight aggregates (EP) and the cement grouting is produced at a microscopic level. To clarify how this interaction affects the thermal resistance and the conductivity, scanning electron microscope (SEM) tests have been made to clarify the interaction between the EP and the cement grouting.

**Figure 6:**
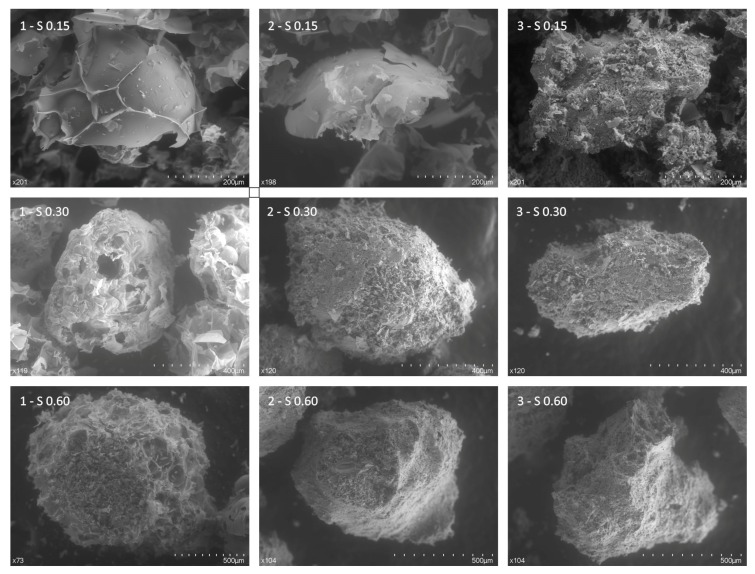
SEM image of expanded perlite size 0.15 mm (1-S0.15 and 2-S0.15), 0.30 mm (1-S0.30), and 0.60 mm (1-S0.60), and cement interface for 0.15 mm (3-S0.15), 0.30 mm (2-S0.30 and 3-S0.30), and 0.60 mm (2-S0.60 and 3-S0.60). A high-resolution version has been included in the Supplementary Materials (Figures S1–S9).

The use of lightweight aggregates in mortars and concretes implies a reduction in the densities and thermal conductivities of these products, as well as a reduction in their mechanical strengths. However, the highly porous foamy microstructure of the EP has different behaviors with the cement grouting in the mortar depending on the particle size, which form closed porosities if the aggregate has a suitable size. As can be seen in Figure 6, the EP in images 1-S0.15 and 2-S0.15 show that the natural perlite with a granulometry of 0.15 mm is formed by partial and not complete microspheres; therefore, it does not have closed pores like those it could have from 0.30 mm (1-S0.30 and 1-S0.06). Likewise, images 3-S0.15, 2-S0.30, 3-S0.30 (EP with cement grouting) show that the cement grouting has micro-scales that could have been produced by the grinding of the aggregate in the mixing and compacting processes, where the initial granulometries would have been reduced, losing part of their initial closed porosity.

**Figure 7:**
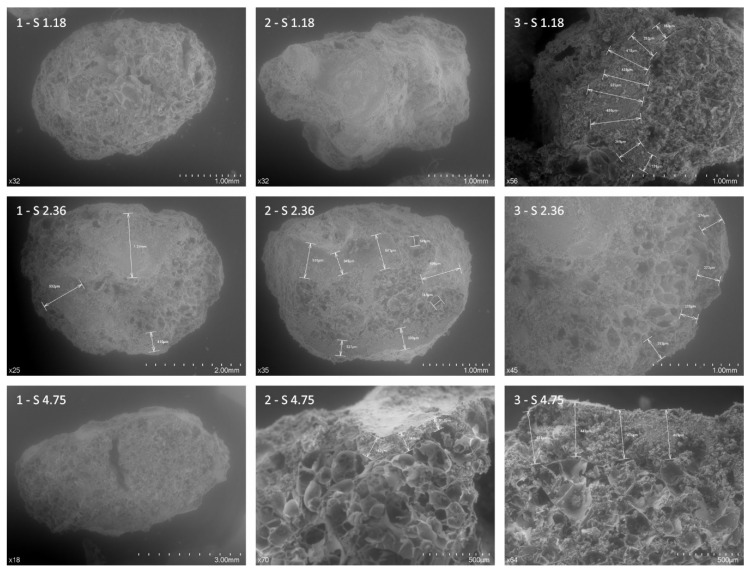
SEM image of expanded perlite size 1.18 mm (1-S1.18) and cement interface for 1.18 mm (2-S1.18 and 3-S1.18), 2.36 mm (1-S2.36, 2-S2.36; and 3-S2.36) and 4.75 mm (1-S4.75, 2-S4.75, and 3-S4.75). A high-resolution version has been included in the Supplementary Materials (Figures S10–S18).

**Figure 8:**
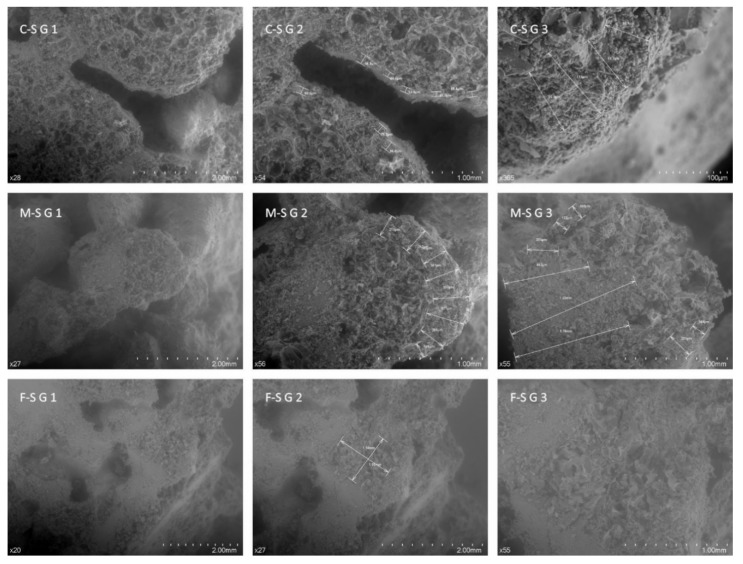
SEM image of expanded perlite and cement interface at C-SG, M-SG, and F-SG (C-SG1, C-SG2, C-SG3; M-SG1, M-SG2, M-SG3; F-SG1, F-SG2, and F-SG3). A high-resolution version has been included in the Supplementary Materials (Figures S19–S27).

Aggregates with the largest sized granulometry (1.18–4.75) (Figure 7) show how the cement grouting completely covers the surface of the aggregates (2-S1.18 and 1-S4.75); however, those images with cross-sectional cuts depict the cement grouting’s penetration face (3-S1.18, 1-S2.36, 2-S2.36, 3-S2.36, 2-S4.75, and 3-S4.75). It can be seen that the penetration is not homogeneous, it depends on the aggregate’s capillary network and ranges between 26 and 1320 nm, with an average minimum value of 111 nm, a maximum value of 758 nm, and an average mean penetration of 322 nm (Figure 7 and Figure 8). This implies an important reduction of the insulating capacity of the aggregate, with this reduction increasing the smaller the particle size is (Figure 8, F-SG2). Therefore, the reduction of the perlite’s foamy microstructure for very fine particles would be 100% for sizes between 0.15 and 0.6 mm, if the grout absorption is between 322 and 758 nm and between 75% and approximately 98%, if the mean penetration is 111 nm. This implies that only the coarse 1.18 and 2.36 granulometries would keep the majority of their porosity, finding a reduction of between 56% and 26% respectively for 111 nm, and between 91% and 62% for 322 nm. However, if the penetration of the grouting were greater, considering the average maximum value measured of 758 nm, only aggregates measuring over 2.36 mm would conserve approximately 5% of their grout free porous structure.

## 4. Discussion

Despite fineness modulus complying with the manufacturing standards [5], the manipulation of the granulometry, without additional processes like grinding or the pre-water absorption, has shown substantial variations of the thermal conductivity (−45%), density (−43%), and water absorption (−57%). This can be considered beneficial as the material obtained is a better insulator, lighter, and less porous. On the other hand, the compressive strength, starting at already low values, has been reduced by 69%. 

The results suggest that there is a complex relation between the size of lightweight aggregates and other characteristics of the resulting mortar: The density, compressive strength, conductivity, and water absorption. For this reason, it is not clear that there is an evident and direct relation between the independent variable of the study and each one of the dependent variables. However, it does seem clear that their variation affects each one of these, in one way or another. The reason, according to the conclusions of this study, along with other similar ones, may be the combined effect of the physical and chemical characteristics of the EP that are, in turn, a result of the particle sizes. Its internal porous structure generates considerable variations in the mortar’s density, the voids between particles have an effect on the water absorption and, the combination of both affect the resulting materials’ compressive strength. Each one of these aspects is analyzed in more detail below.

The densities obtained in this study, using exclusively EP as a lightweight aggregate are between 400 and 1000 kg/m^3^. These values are in line with those obtained by other authors. Sengul et al. [4] obtained values between 2000 and 400 kg/m^3^, depending on the percentage of EP substituting the natural aggregate; however in their study, EP was only used with a size between 2 and 4 mm, while in this research, the size has always been below 2.36 mm. Other authors have obtained densities between 1350 and 2000 kg/m^3^ using different lightweight aggregates with fineness modules between 3.40 and 4.28 [5]. The results obtained suggest that, for small fineness modulus, there may be a linear correlation between these and the mixture’s density, something which has not been suggested by other authors. 

The relation between density and compressive strength has been studied in more depth for different types of lightweight aggregates. Some authors suggest a slightly quadratic relation for densities between 400 and 2000 kg/m^3^ [4], although this study has approached a linear relation. However, it also mentions that when the EP replaces more than 40% of the fine aggregate, the effect on the compressive strength is more evident. The results of this study suggest the same, approaching a linear relation, considering that, in this case, the replacement of the aggregate for EP is always at 100%. This must be considered, since it cannot be generalized for mixtures of different lightweight aggregates. 

The compressive strength values are very low (never higher than 0.5 MPa). However, considering that in this case, the fine aggregate replacement is 100%, the values are similar to those obtained by other authors for similar densities [56], although in other cases there was not a complete replacement of the fine aggregate and they added additives to the mixture. We speak, therefore, about a mortar that cannot be used for structural purposes, but that can be used for others, like the coating of facades, since it falls within the UNE-EN 998-1:2010 category for coating mortars in the CS1 category (compressive strength requirements between 0.4–2.5 N/mm^2^) [57]. Therefore, by using EP exclusively, low mechanical strengths are obtained, but by varying the fineness modulus, the strength can be increased by up to 323%; this relation seems linear. 

The thermal conductivities obtained are pretty low, at between 0.18 and 0.10. Other studies, for similar densities have obtained conductivities between 0.35 and 0.13 [4]; it can also be highlighted that in other cases, even when using EP as a unique lightweight aggregate, the conductivity is reduced to 0.13. Given that in this case, the EP’s granulometry was between 2 and 4 mm, it can be seen that a suitable graduation of the EP’s granulometry can reduce conductivity by 23%. However, these statements must be made with due caution, as the cement content can also have an impact on this parameter. The relation between the fineness modulus and conductivity provides an R^2^ of 0.92, when other studies have validated conductivity and density relations with an R^2^ of 0.94 [8].

Finally, the water absorption percentage is also a parameter that definitely affects the thermal conductivity and it seems to depend, in turn, on the fineness modulus. In this study, these relations seem very clear, linear, and with high R^2^ coefficients. The larger grain size for the EP leads to a drastic reduction of water absorption (58%); meanwhile, as the water absorption falls, so does the thermal conductivity (−44%). This suggests there may be a connection between these. However, even though not many studies have considered this variable, some suggest contradictory results: the higher the water absorption, the lower the thermal conductivity [4].

The scanning electron microscopy explains how the mortar made with larger fineness modulus has a lower thermal conductivity, density, water absorption, and mechanical strength, essentially because the cement grouting does not penetrate the closed pores which the larger sized aggregates hold, with the average penetration of the grout of between 66 and 409 nm, depending on the size of the particle. Because of this, in the small sized aggregates, the capillary network causes their silting by cement grouting to be almost total; that also explains the higher density of these samples, as well as the higher thermal conductivity. However, the smaller size of particles also means higher water absorption, although the open pores are filled with cement; this can be explained by the fact that the capillary network of the cement still absorbs water even after filling the open pores.

## 5. Conclusions

This study set out to determine whether the granulometry of lightweight aggregates, in this case EP, can affect given properties of the mortar. The results support the following conclusions:

- The variation of the EP’s granulometry, when this is used as a unique lightweight aggregate in the preparation of mortar, has a noticeable influence on the density of the resulting product, its thermal conductivity, its compressive strength, and its degree of water absorption.
- The results suggest linear relations among several of the variables studied, which is corroborated by results of similar studies; likewise, the R^2^ coefficients are acceptable for several of the correlations analyzed. Although these results are promising, it is also true that the number of samples is low, and this is one of the main limitations of this study. Therefore, in the future, it would be desirable to test a higher number of samples to be able to validate these findings.
- The SEM images indicate that the particle size, amount of water, use of fluidifier additives, and the mixing and compacting time play a fundamental role in the thermal behavior of this type of mortar, given that a greater fluidity and a higher mixing and compacting time can cause a higher saturation of the crystalline structure of the aggregate, improving the penetration of the cement grouting in the porous structure. However, this must be studied in detail in future research.
- According to the images, it can be concluded that the initial particle size is essential, and that even within the common limits established by construction standards, enormous differences can be found. It might be the case that the use of larger particle sizes offers a higher number of closed pores which are not silted by the cement grouting, providing better thermal and water benefits. Besides, a higher amount of water, the use of fluidifying additives and a longer mixing and compacting time can cause a higher saturation of the aggregate’s crystalline structure, improving the penetration of the cement grouting in the porous structure.
- However, it remains unclear how smaller particle size is related to a higher water absorption. The explanation hereby provided is based on an educated guess, considering the results from all the tests: Cement and water fill the space between particles, giving a mixture with higher density and higher thermal conductivity. However, further research should be conducted to clarify how this void space is filled with both cement and water; the capillary network of the cement might offer an explanation, but, as stated before, research should be conducted to prove this hypothesis.

## Figures and Tables

**Table 1:** Chemical composition of the expanded perlite.

Compound	Perlite
SiO_2_	75.5%
Al_2_O_3_	14.6%
Fe_2_O_3_	0.60%
CaO	0.85%
MgO	0.20%
Na_2_O	2.99%
K_2_O	5.26%
Total	100.00%
Density	0.7 ± 1 g/cc
pH	pH = 7.0–8.0

**Table 2:** Densities for expanded perlite.

Apparent Density				Compacted Density			
Density (kg/m^3^)	Das1	Das2	Das	Density (kg/m^3^)	Dac1	Dac2	Dac
PP8	78	80	79	PP8	88	96	92
PP6	77	75	76	PP6	90	97	94
C-SG	76	76	76	Coarse	98	99	99
M-SG	80	81	81	Medium	98	99	99
F-SG	87	85	86	Fine	110	112	111

**Table 3:** Mix proportions of lightweight mortars.

Code	Cement (kg/m^3^)	Water (L/m^3^)	Water/Cement	Expanded Perlite for Size in mm (kg)								EP Fineness Modulus
				4.75	2.36	1.18	0.6	0.3	0.15	R	Total	
F-SG	170	228	1.34	0	0	12.15	20.25	28.35	12.15	8.1	81	2.20
M-SG	170	228	1.34	0	5.67	24.3	18.63	20.25	8.1	4.05	81	2.85
C-SG	170	228	1.34	0	12.15	36.45	16.2	12.15	4.05	0	81	3.50

**Table 4:** Laboratory test results.

Code	Volume (cm^3^)	Humidity State Mass (gr)	Dry State Mass (gr)	Humidity State Density (kg/m^3^)	Dry State Density (kg/m^3^)	Thermal Conductivity (W/m·K)	Compressive Strength (MPa)	Water Absorption (%)
F-SG	2.870	2895.4	1896.4	1008.8	660.8	0.184	0.42	158
M-SG	3.037	2129.2	1225.4	720.0	403.5	0.163	0.21	137
C-SG	3.032	2104.1	1138.1	540.1	375.4	0.100	0.13	67

## Supplementary Materials

The following are available online at https://www.mdpi.com/1996-1944/12/23/4013/s1, Figure S1: 1-S0.15—SEM image of expanded perlite size 0.15 mm; Figure S2: 2-S0.15—SEM image of expanded perlite size 0.15 mm; Figure S3: 3-S0.15—SEM image of cement interface for 0.15 mm; Figure S4: 1-S0.30—SEM image of expanded perlite size 0.30 mm; Figure S5: 2-S0.30—SEM image of cement interface for 0.30 mm; Figure S6: 3-S0.30—SEM image of cement interface for 0.30 mm; Figure S7: 1-S0.60—SEM image of expanded perlite size 0.60 mm; Figure S8: 2-S0.60—SEM image of cement interface for 0.60 mm; Figure S9: 3-S0.60—SEM image of cement interface for 0.60 mm; Figure S10: 1-S1.18—SEM image of expanded perlite size 1.18 mm; Figure S11: 2-S 1.18—SEM image of cement interface for 1.18 mm; Figure S12: 3-S 1.18—SEM image of cement interface for 1.18 mm; Figure S13: 1-S2.36—SEM image of cement interface for 2.36 mm; Figure S14: 2-S 2.36—SEM image of cement interface for 2.36 mm; Figure S15: 3-S 2.36—SEM image of cement interface for 2.36 mm; Figure S16: 1-S4.75—SEM image of cement interface for 4.75 mm; Figure S17: 2-S 4.75—SEM image of cement interface for 4.75 mm; Figure S18: 3-S 4.75—SEM image of cement interface for 4.75 mm; Figure S19: C-SG1—SEM image of expanded perlite and cement interface at C-SG; Figure S20: C-SG2—SEM image of expanded perlite and cement interface at C-SG; Figure S21: C-SG3—SEM image of expanded perlite and cement interface at C-SG; Figure S22: M-SG1—SEM image of expanded perlite and cement interface at M-SG; Figure S23: M-SG2—SEM image of expanded perlite and cement interface at M-SG; Figure S24: M-SG3—SEM image of expanded perlite and cement interface at M-SG; Figure S25: F-SG1—SEM image of expanded perlite and cement interface at F-SG; Figure S26: F-SG2—SEM image of expanded perlite and cement interface at F-SG; Figure S27: F-SG3—SEM image of expanded perlite and cement interface at F-SG.
