# Supplementary material for: Influence of Granulometry on Thermal and Mechanical Properties of Cement Mortars Containing Expanded Perlite as a Lightweight Aggregate
Source: Materials (Basel). 2019 Dec 3;12(23):4013. doi: 10.3390/ma12234013 (PMC6926826; doi:10.3390/ma12234013)
Supplement: Supplementary file 1 [file materials-12-04013-s001.pdf]

materials

Supplementary Materials: Influence of Granulometry on Thermal and Mechanical Properties of Cement Mortars Containing Expanded Perlite as a Lightweight Aggregate

Matías Leyton-Vergara [1], Alexis Pérez-Fargallo [1], Jesús Pulido-Arcas [1], Galo Cárdenas-Triviño [2] and Jeremy Piggot-Navarrete [3,*]

1 Department of Building Science, University of Bio-Bio, Concepción, 4030000, Chile.
2 Department of Wood Engineering, University of Bio-Bio, Concepción, 4030000, Chile.
3 Department of Design and Theory of Architecture, University of Bio-Bio, Concepción, 4030000, Chile.
* Correspondence: jpiggot@ubiobio.cl

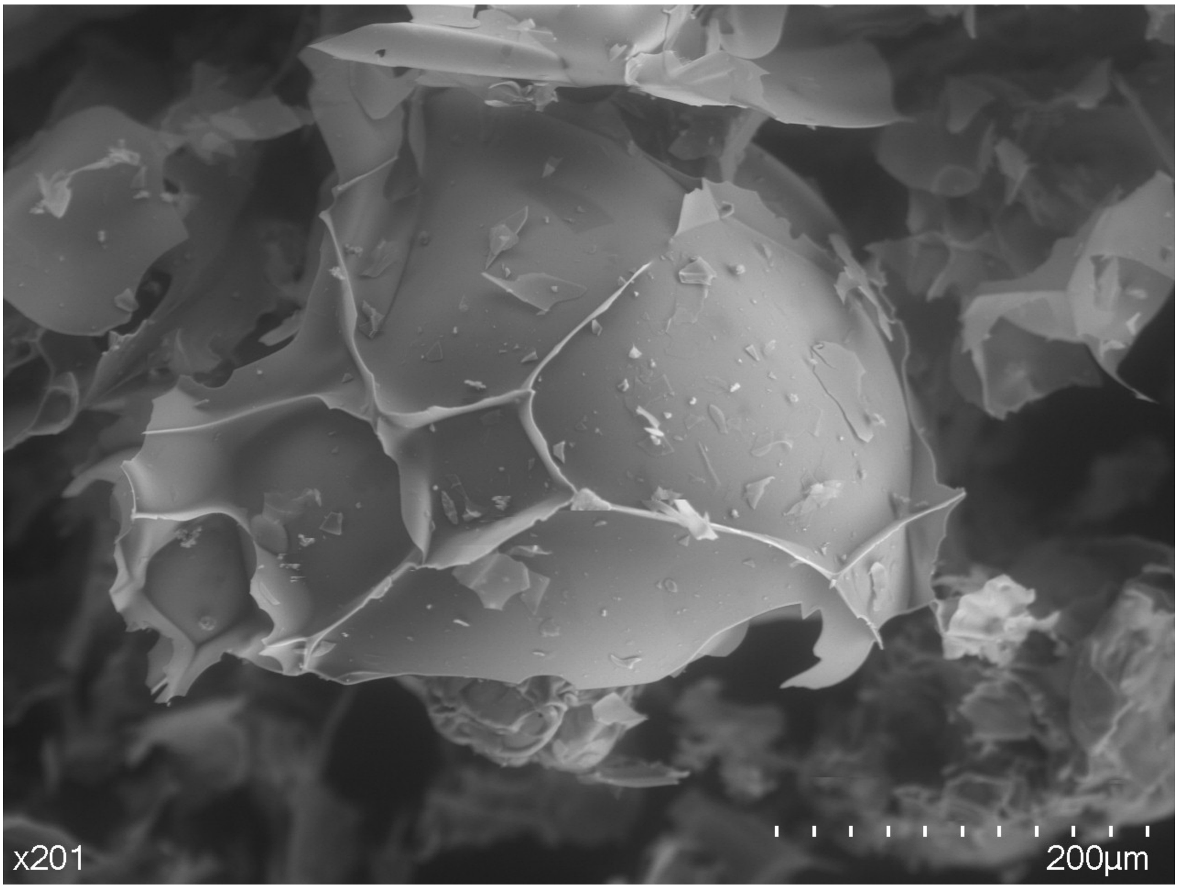

Figure S1. 1-S0.15—SEM image of expanded perlite size 0.15 mm.

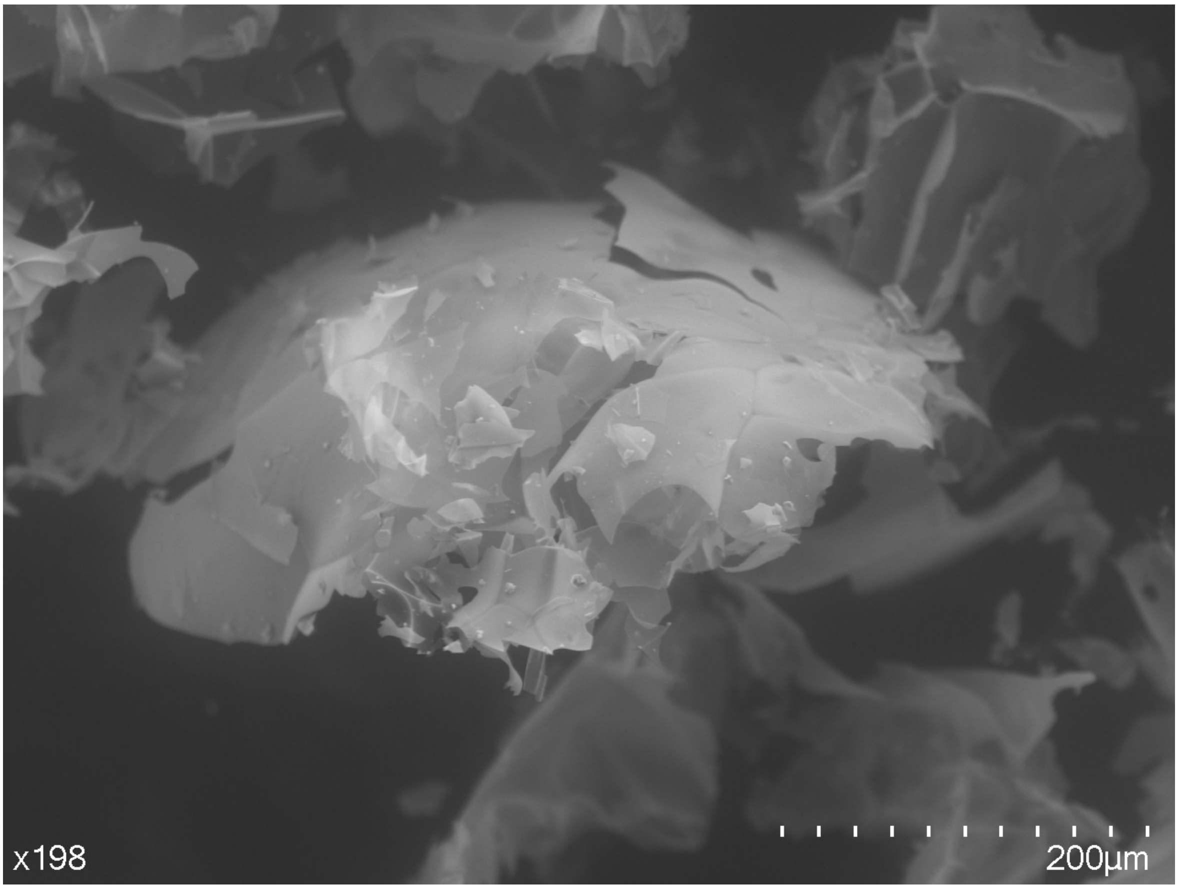

Figure S2. 2-S0.15—SEM image of expanded perlite size 0.15 mm.

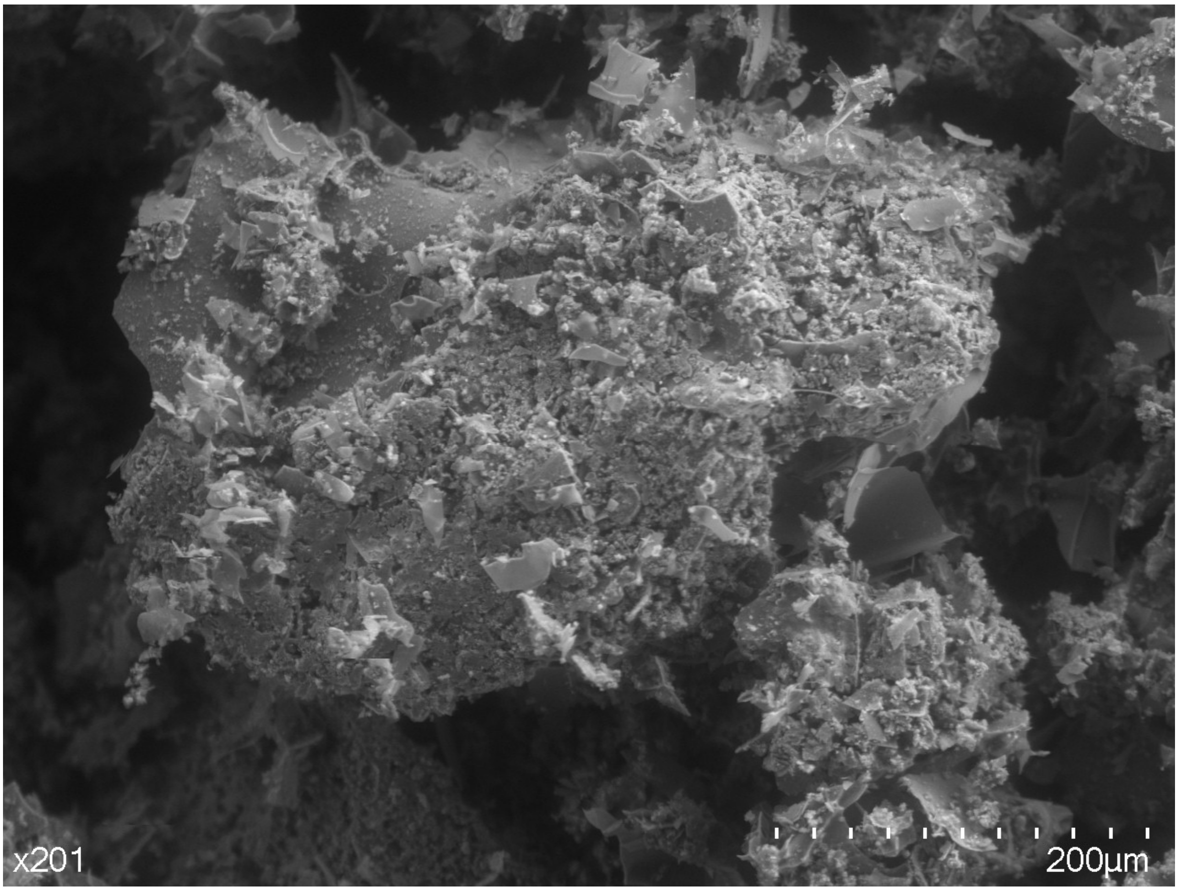

Figure S3. 3-S0.15—SEM image of cement interface for 0.15 mm.

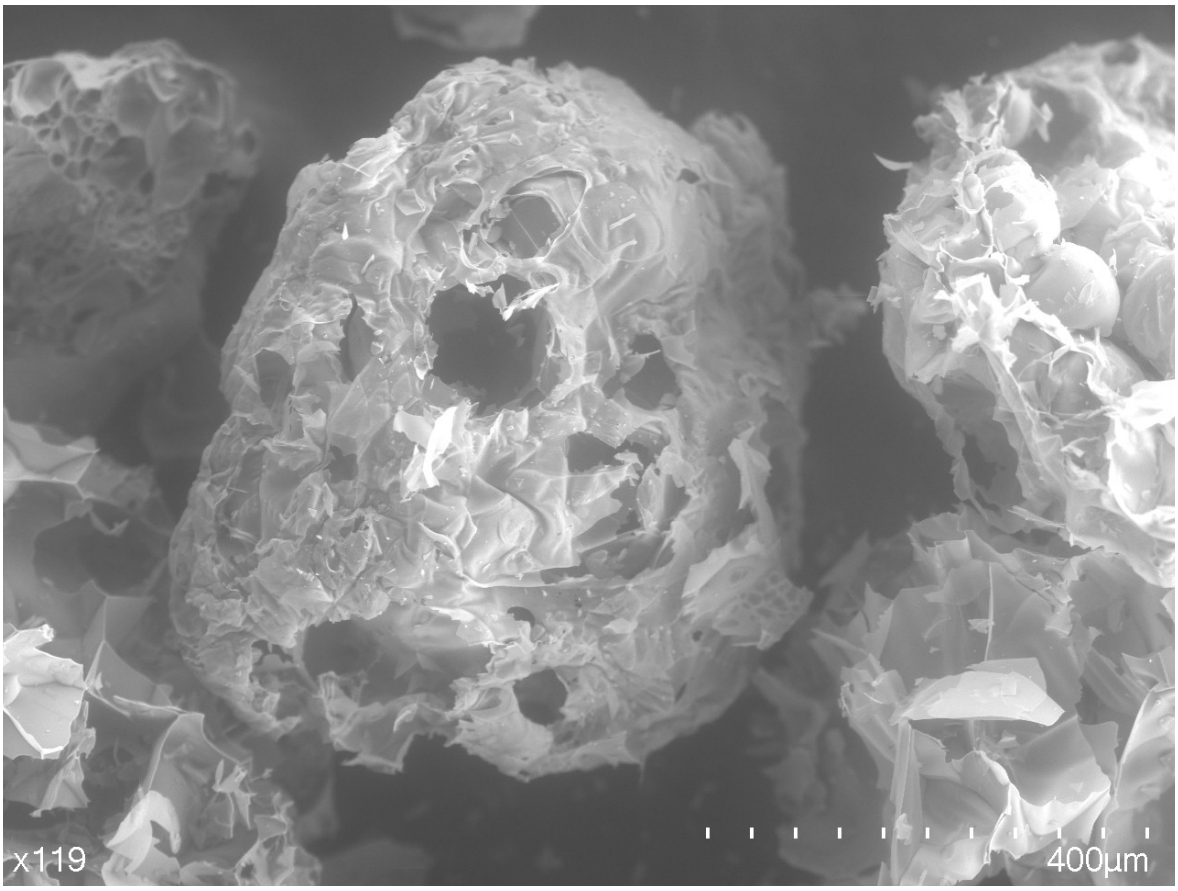

Figure S4. 1-S0.30—SEM image of expanded perlite size 0.30 mm.

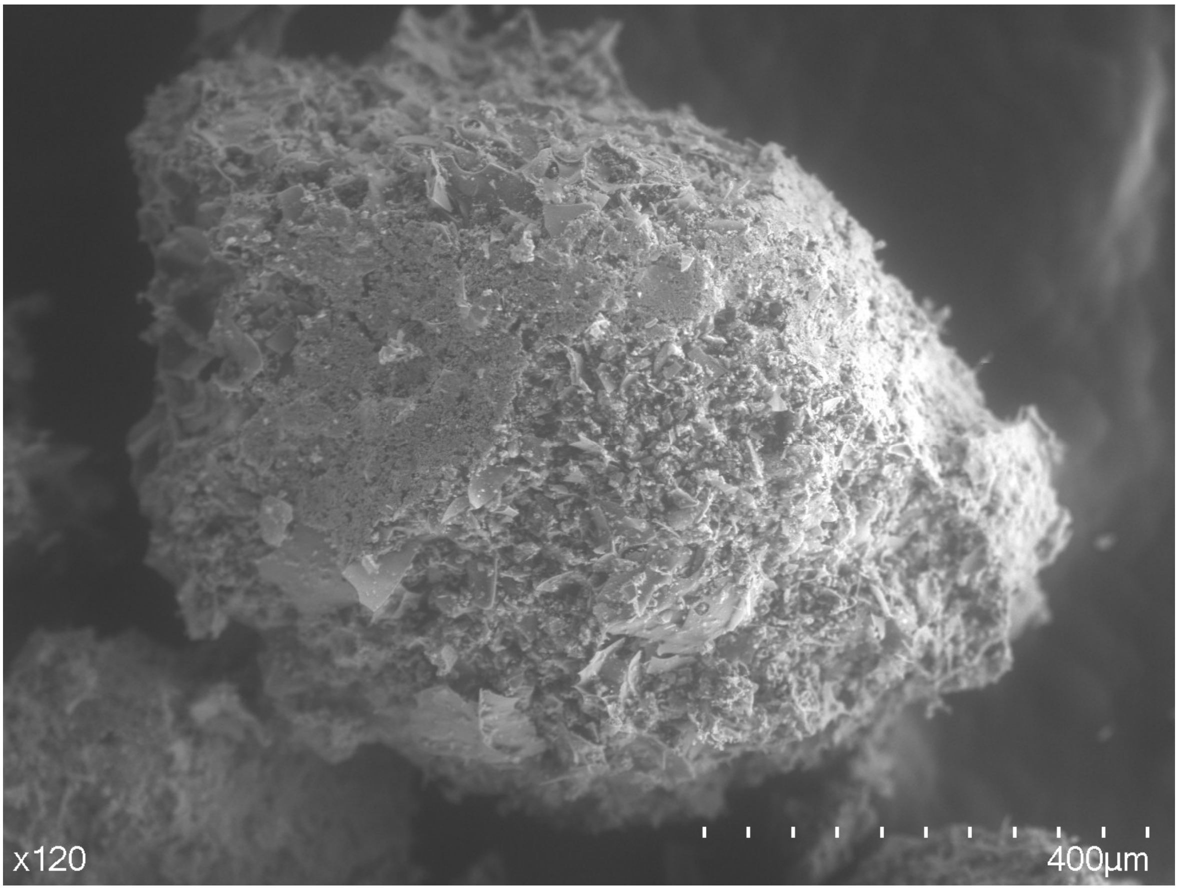

Figure S5. 2-S0.30—SEM image of cement interface for 0.30 mm.

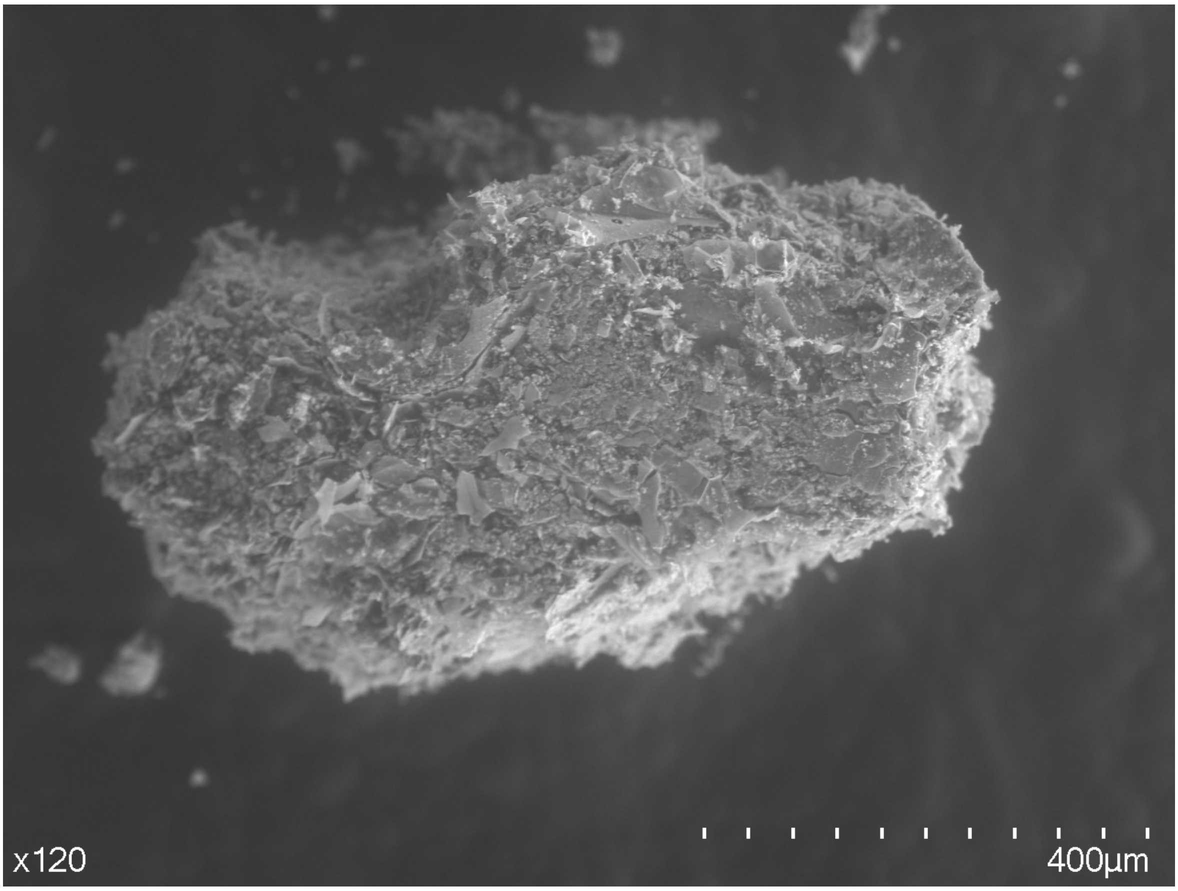

Figure S6. 3-S0.30—SEM image of cement interface for 0.30 mm.

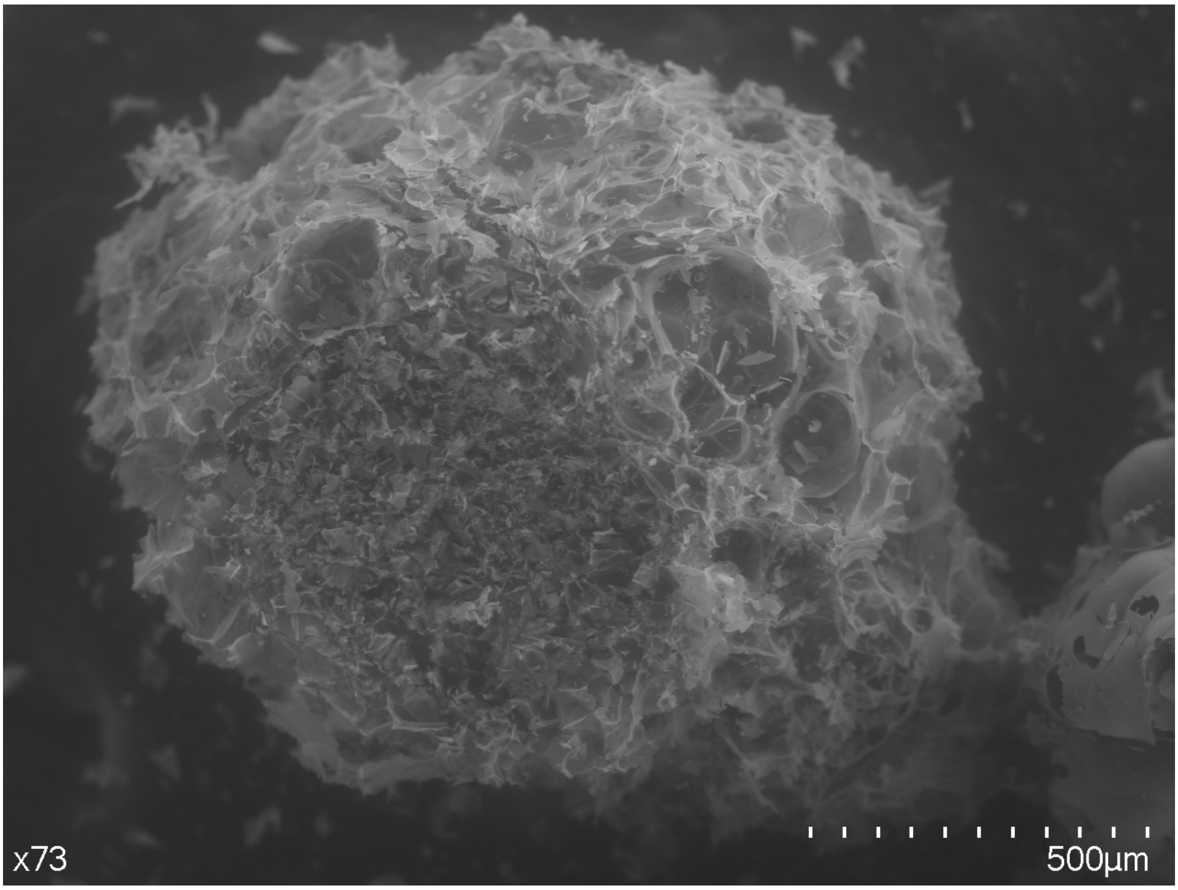

Figure S7. 1-S0.60—SEM image of expanded perlite size 0.60 mm

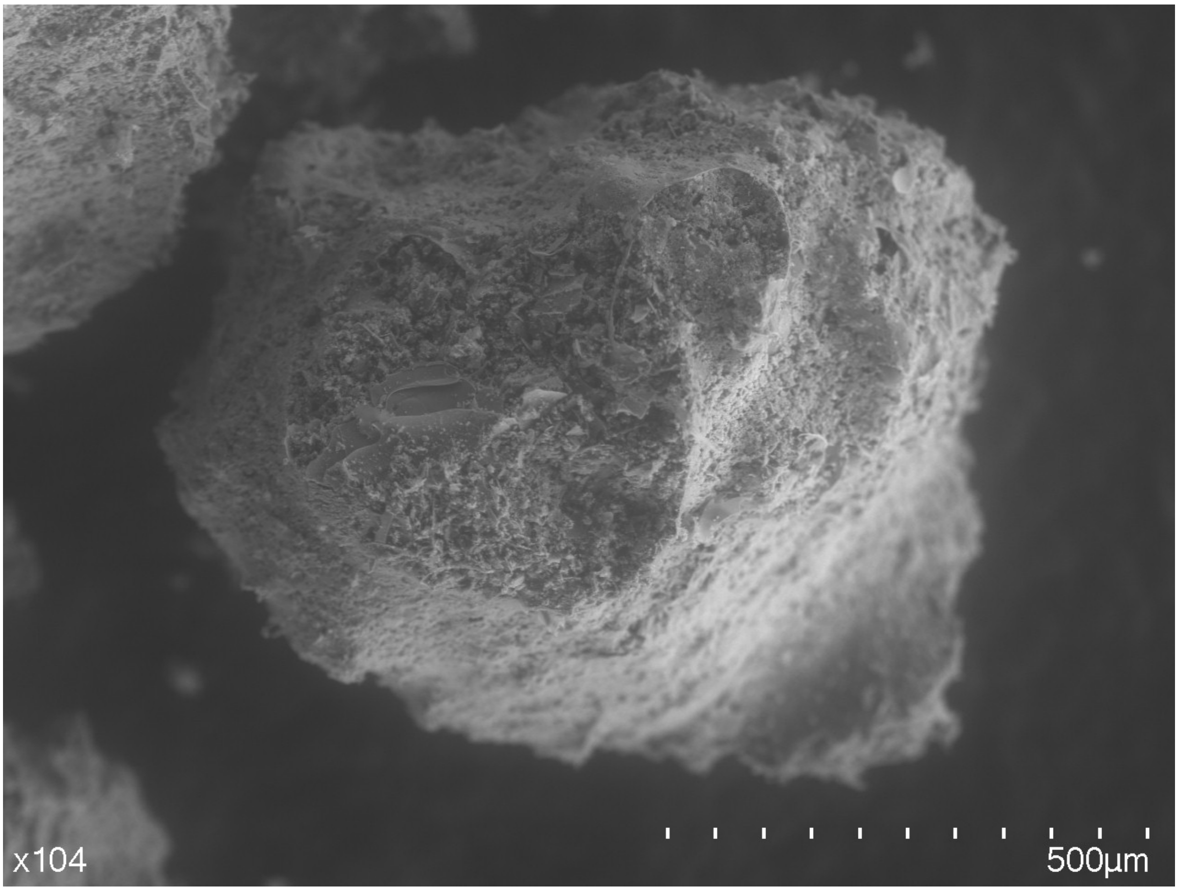

Figure S8. 2-S0.60—SEM image of cement interface for 0.60 mm.

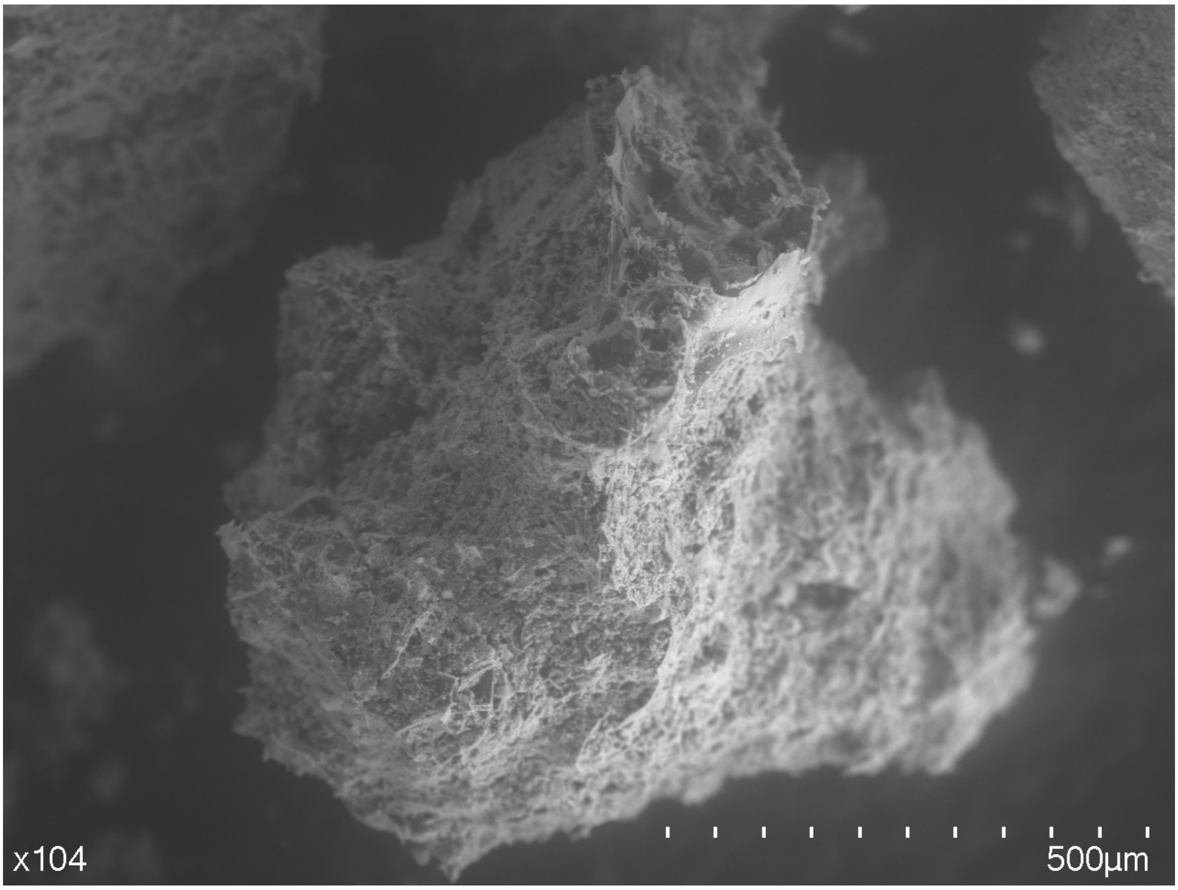

Figure S9. 3-S0.60—SEM image of cement interface for 0.60 mm.

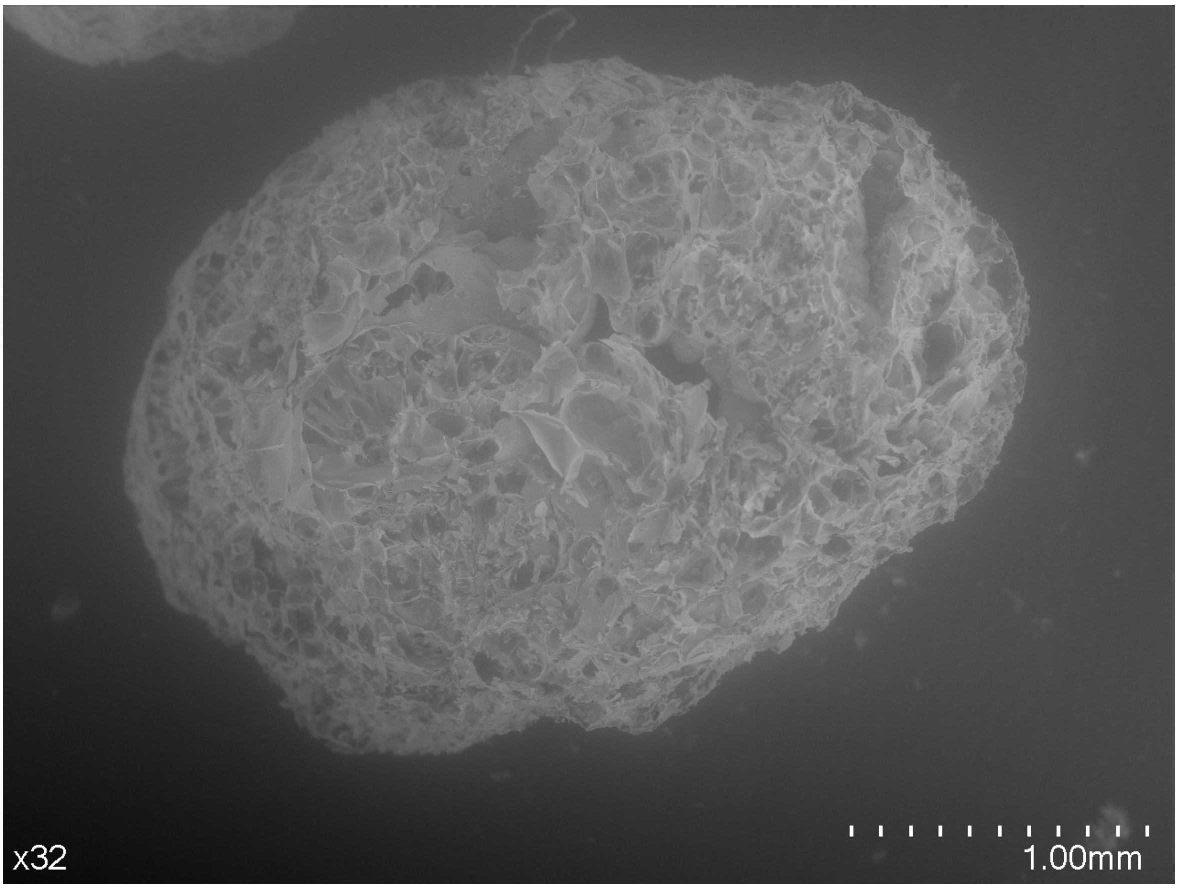

Figure S10. 1-S1.18—SEM image of expanded perlite size 1.18 mm.

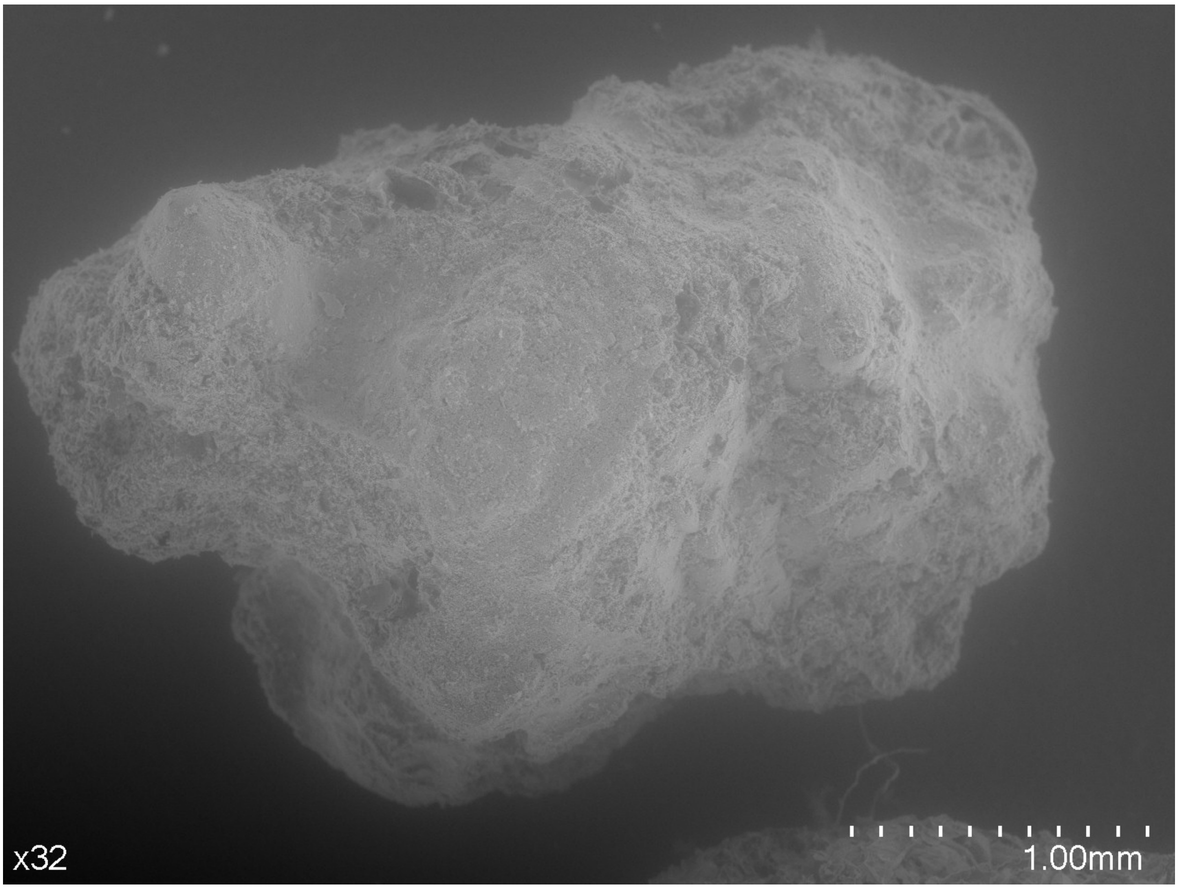

Figure S11. 2-S1.18—SEM image of cement interface for 1.18 mm.

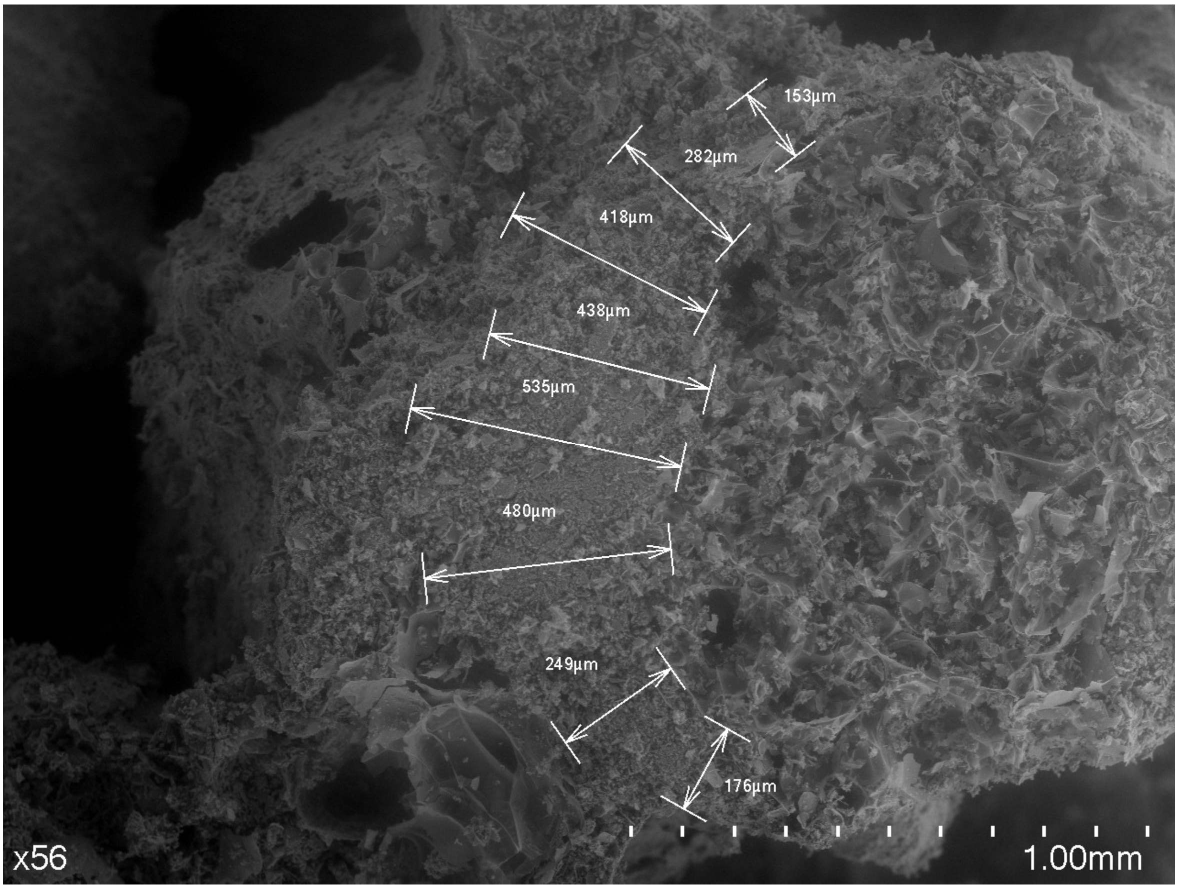

Figure S12. 3-S1.18—SEM image of cement interface for 1.18 mm.

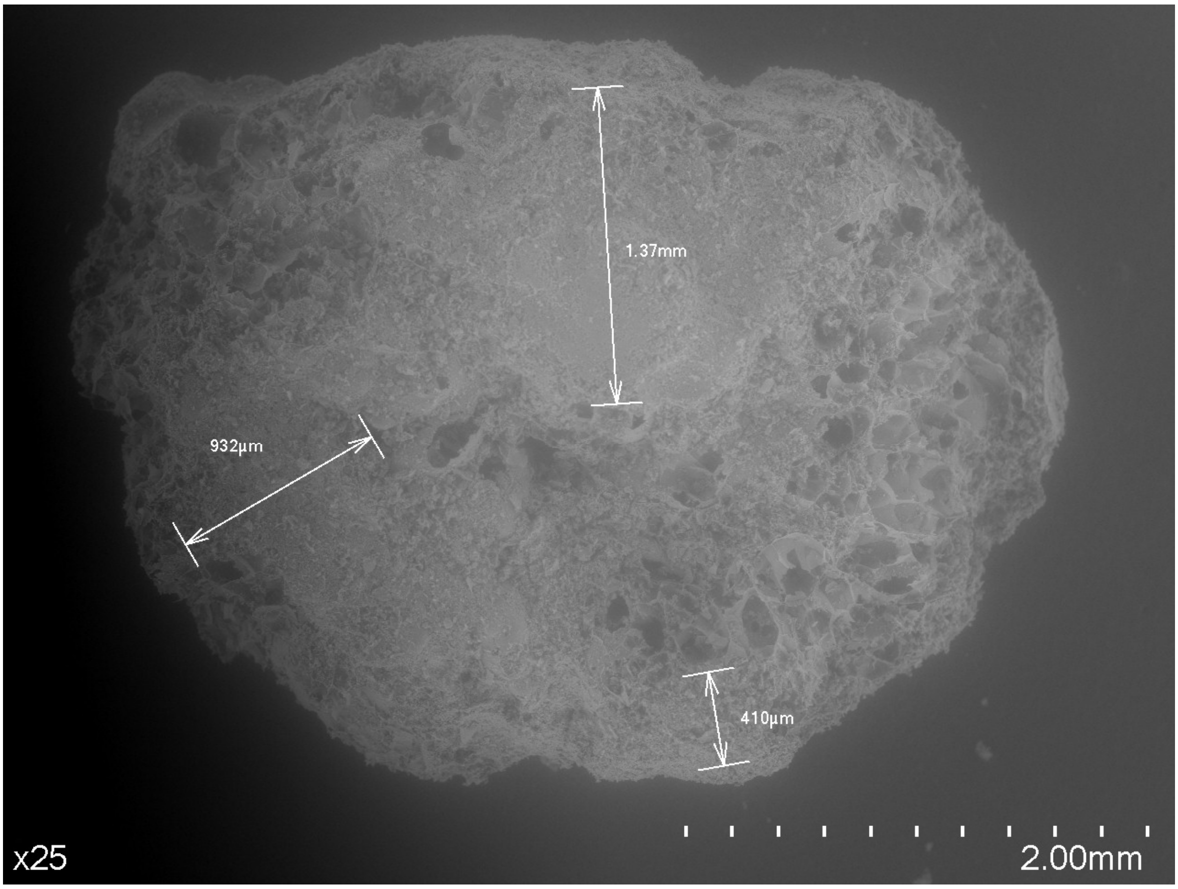

Figure S13. 1-S2.36—SEM image of cement interface for 2.36 mm.

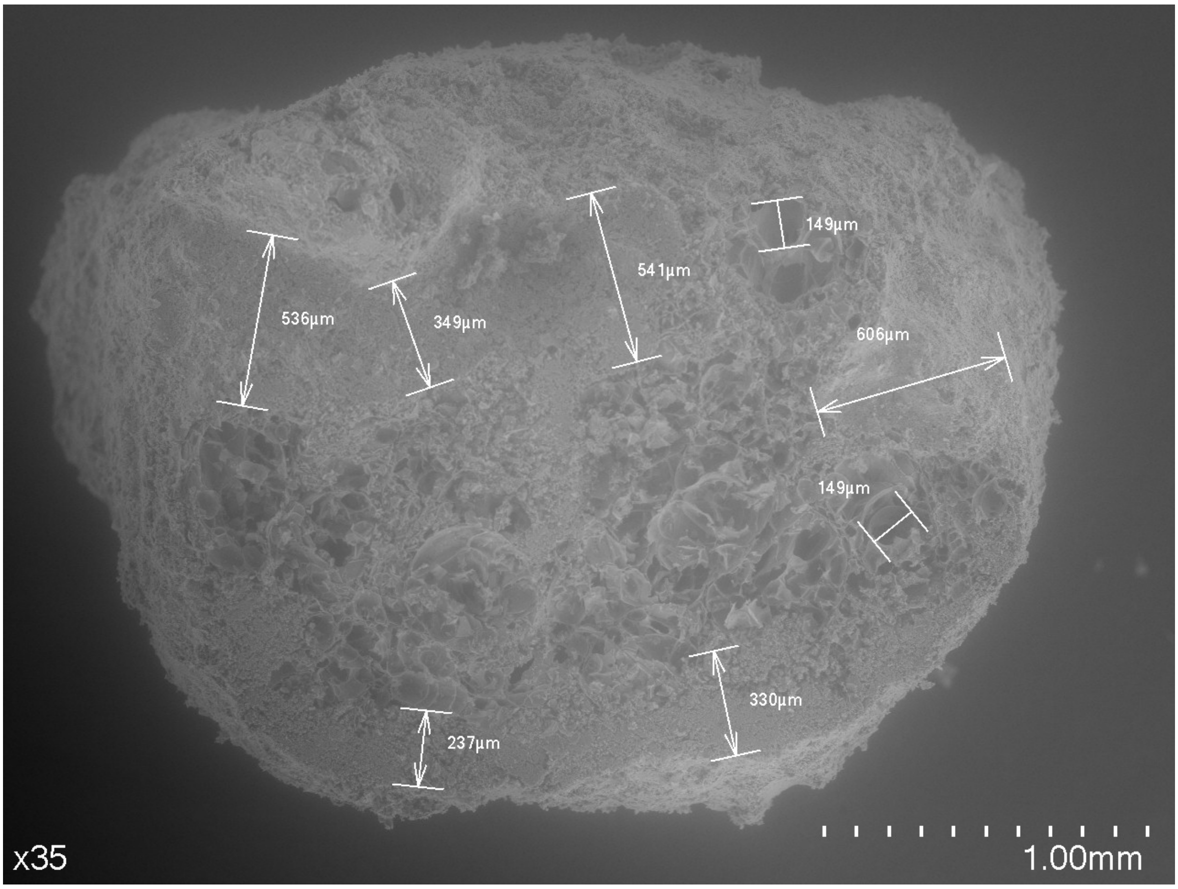

Figure S14. 2-S2.36—SEM image of cement interface for 2.36 mm.

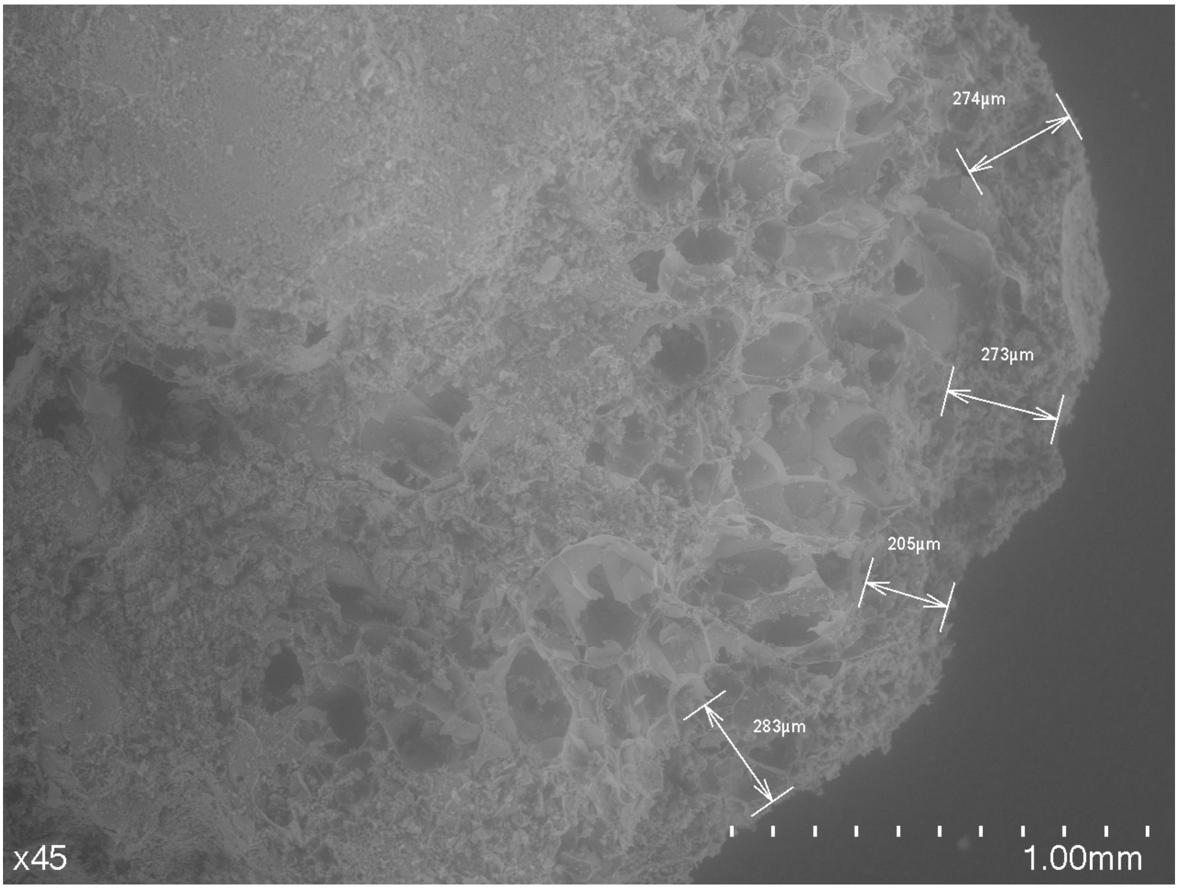

Figure S15. 3-S2.36—SEM image of cement interface for 2.36 mm.

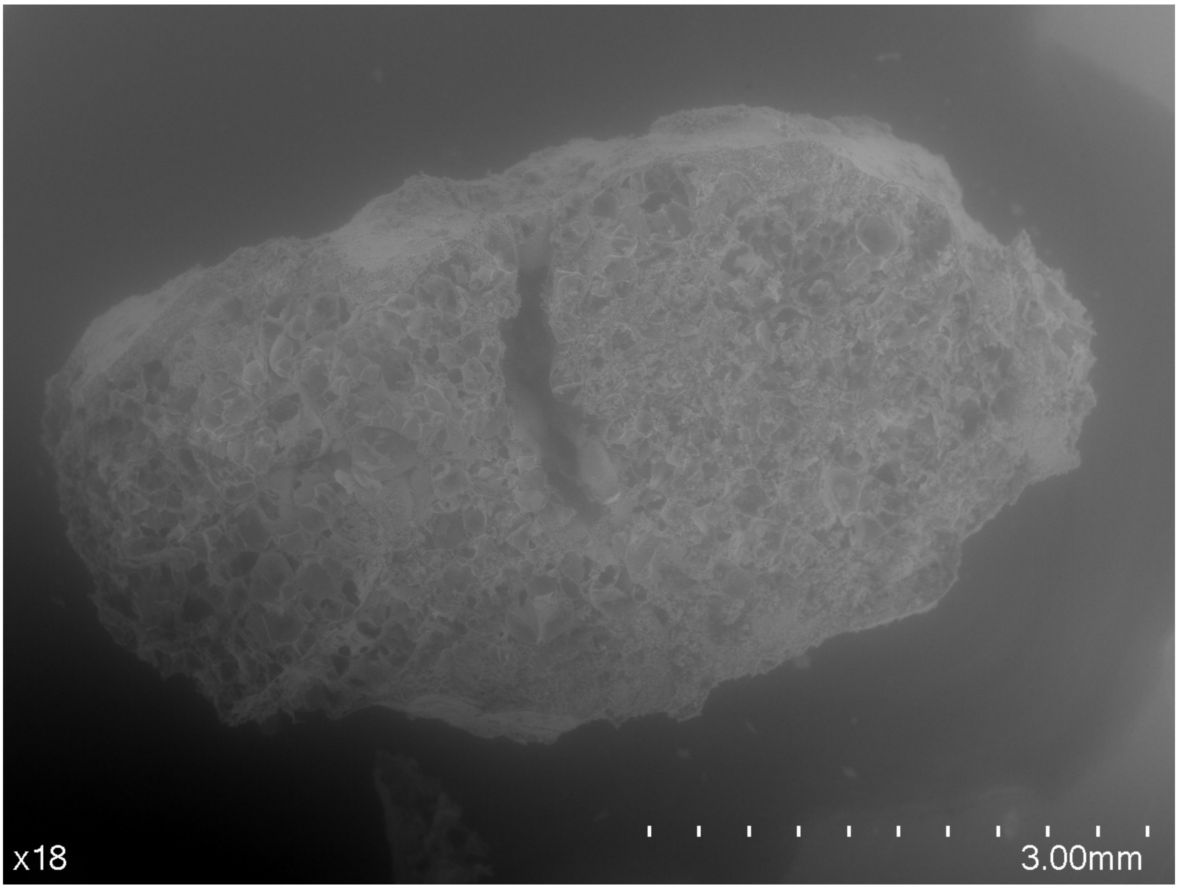

Figure S16. 1-S4.75—SEM image of cement interface for 4.75 mm.

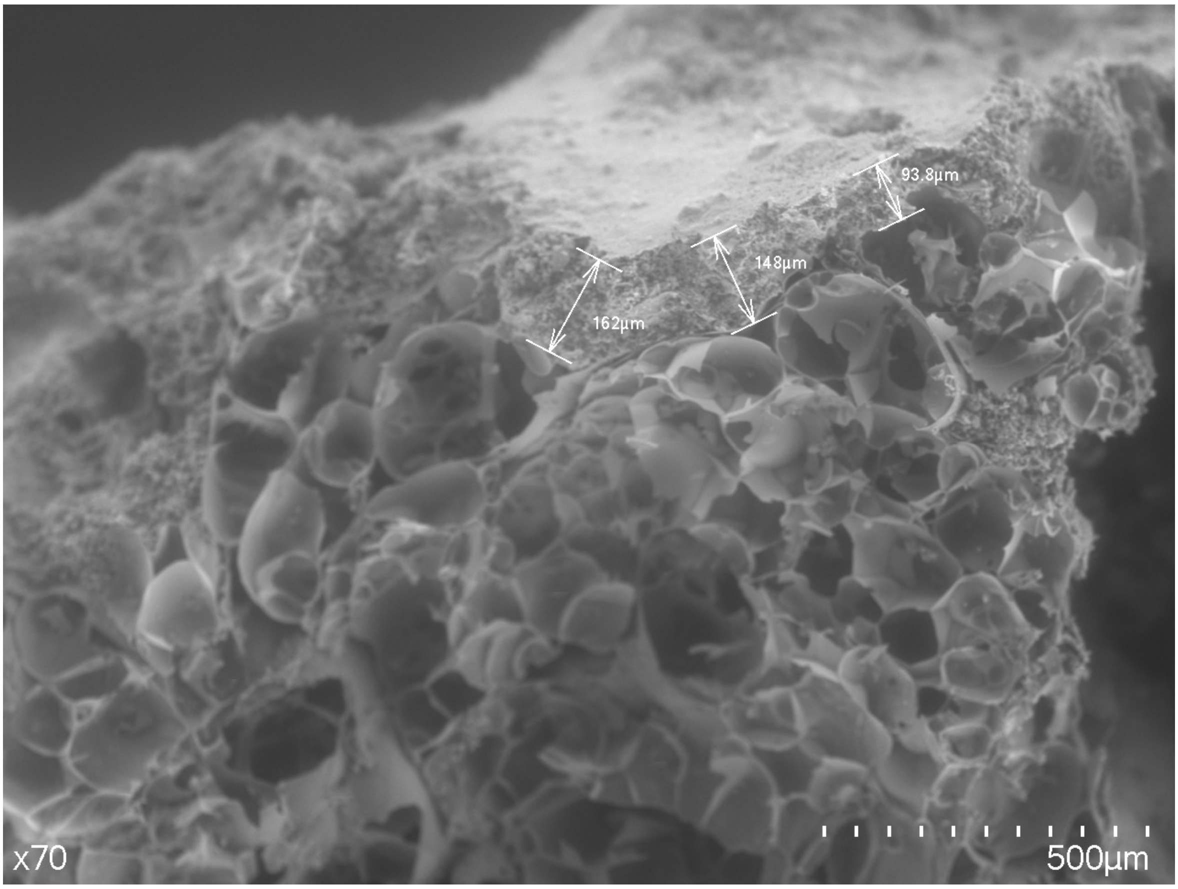

Figure S17. 2-S4.75—SEM image of cement interface for 4.75 mm.

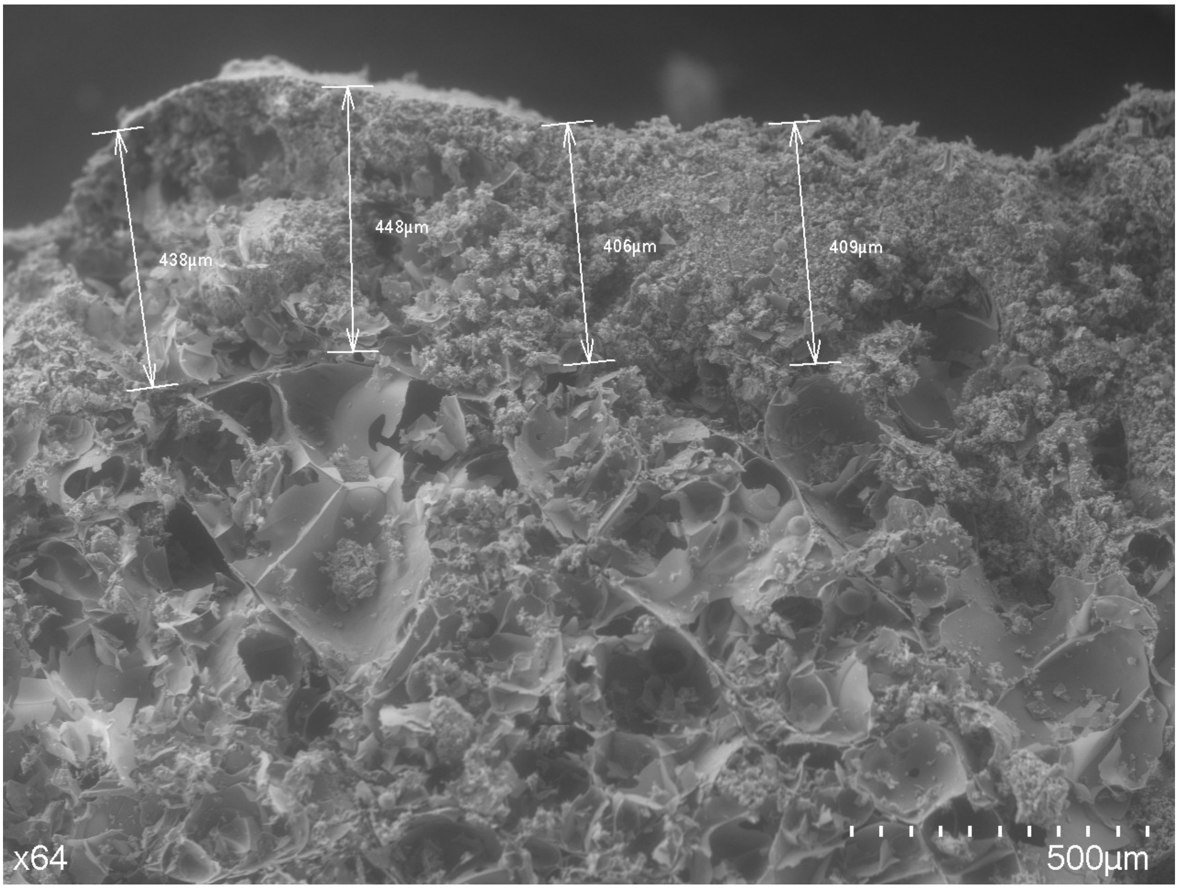

Figure S18. 3-S4.75—SEM image of cement interface for 4.75 mm.

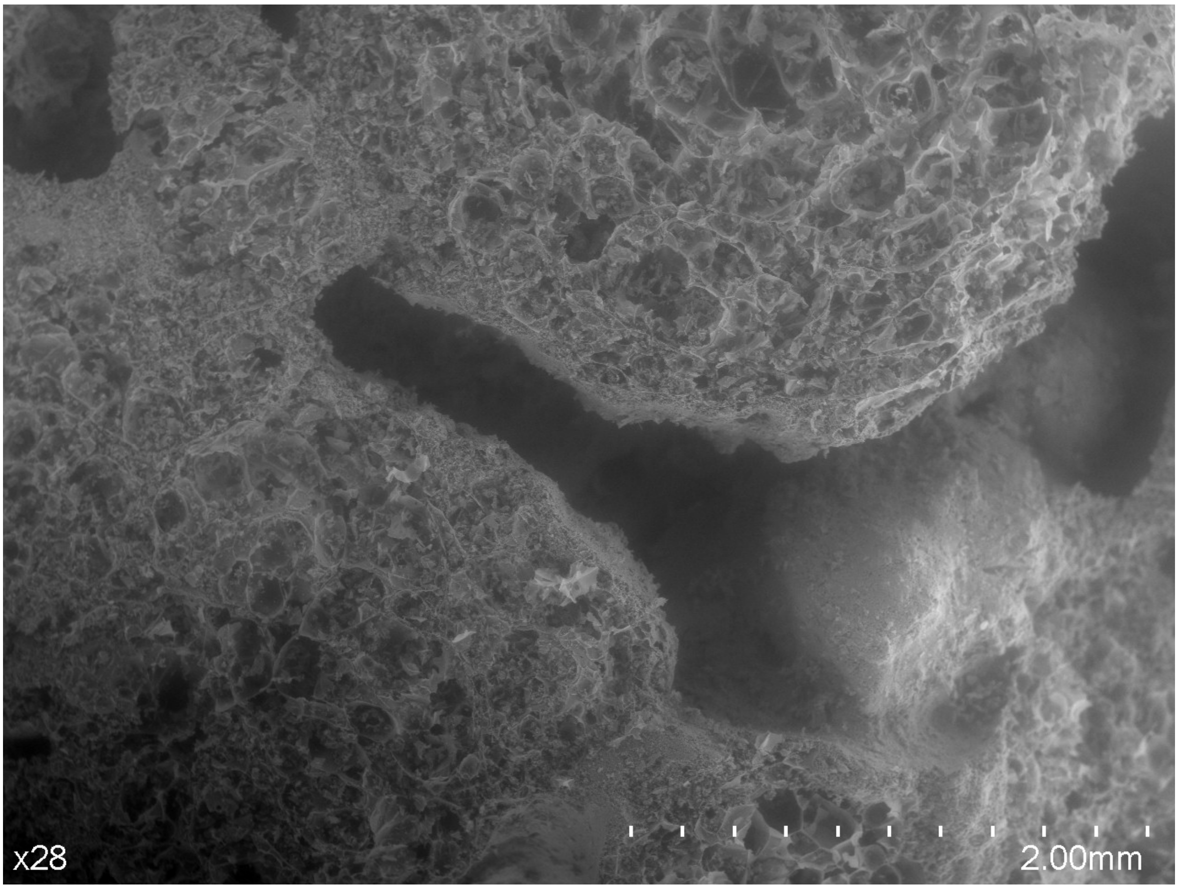

Figure S19. C-SG1—SEM image of expanded perlite and cement interface at C-SG.

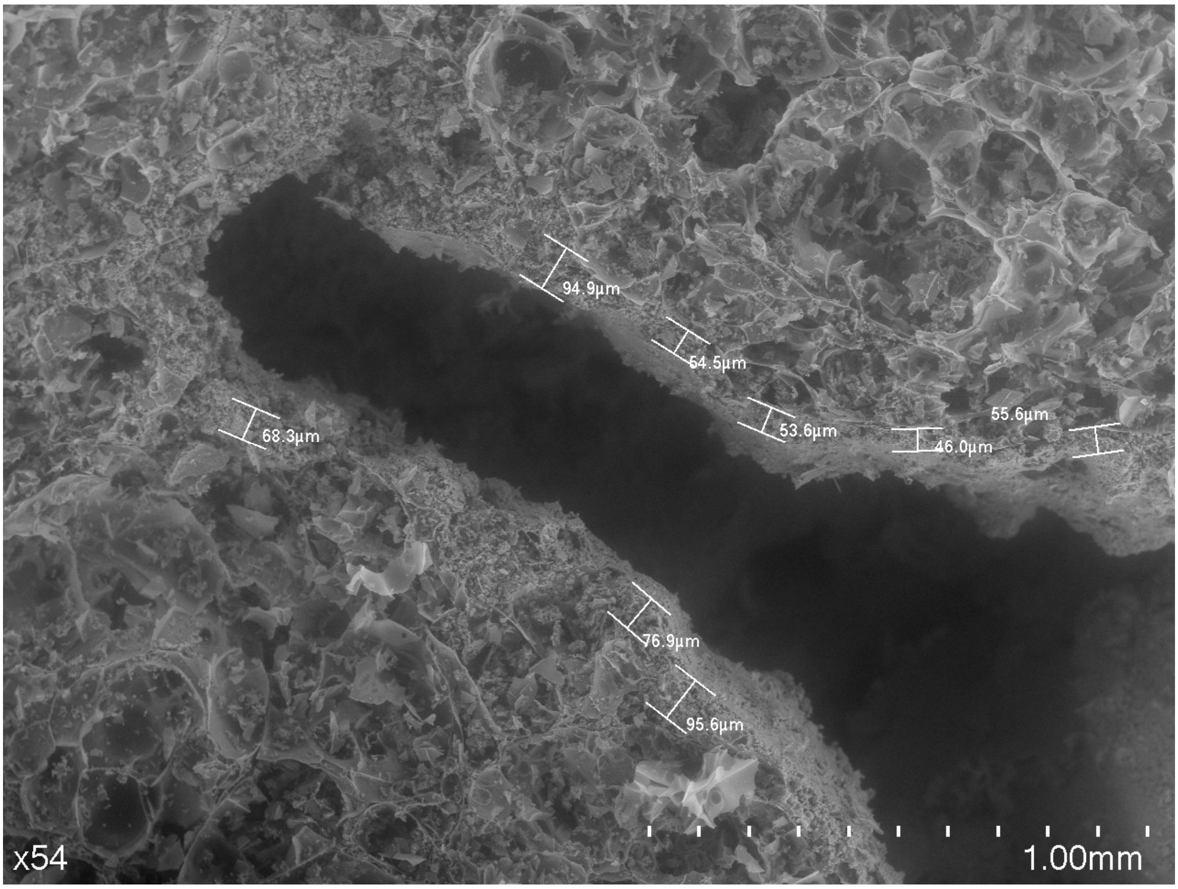

Figure S20. C-SG2—SEM image of expanded perlite and cement interface at C-SG.

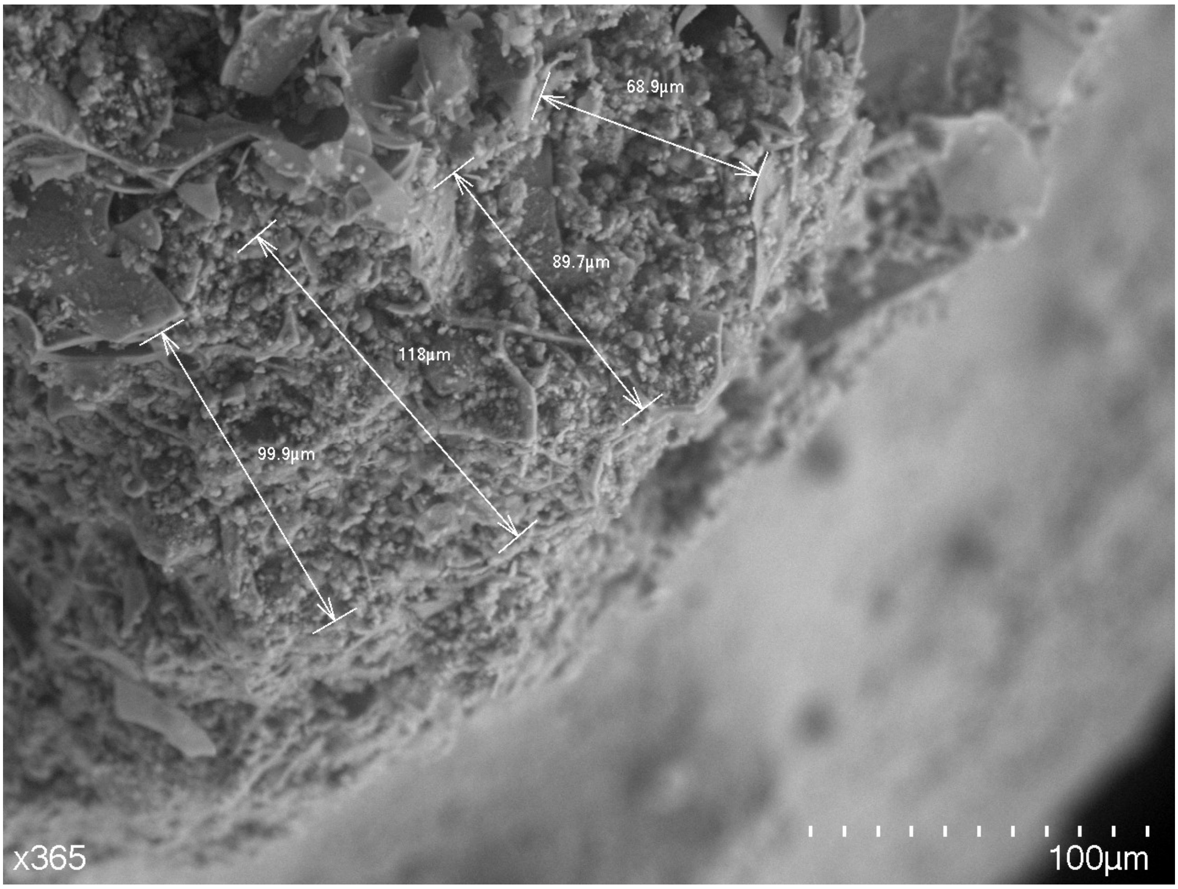

Figure S21. C-SG3—SEM image of expanded perlite and cement interface at C-SG.

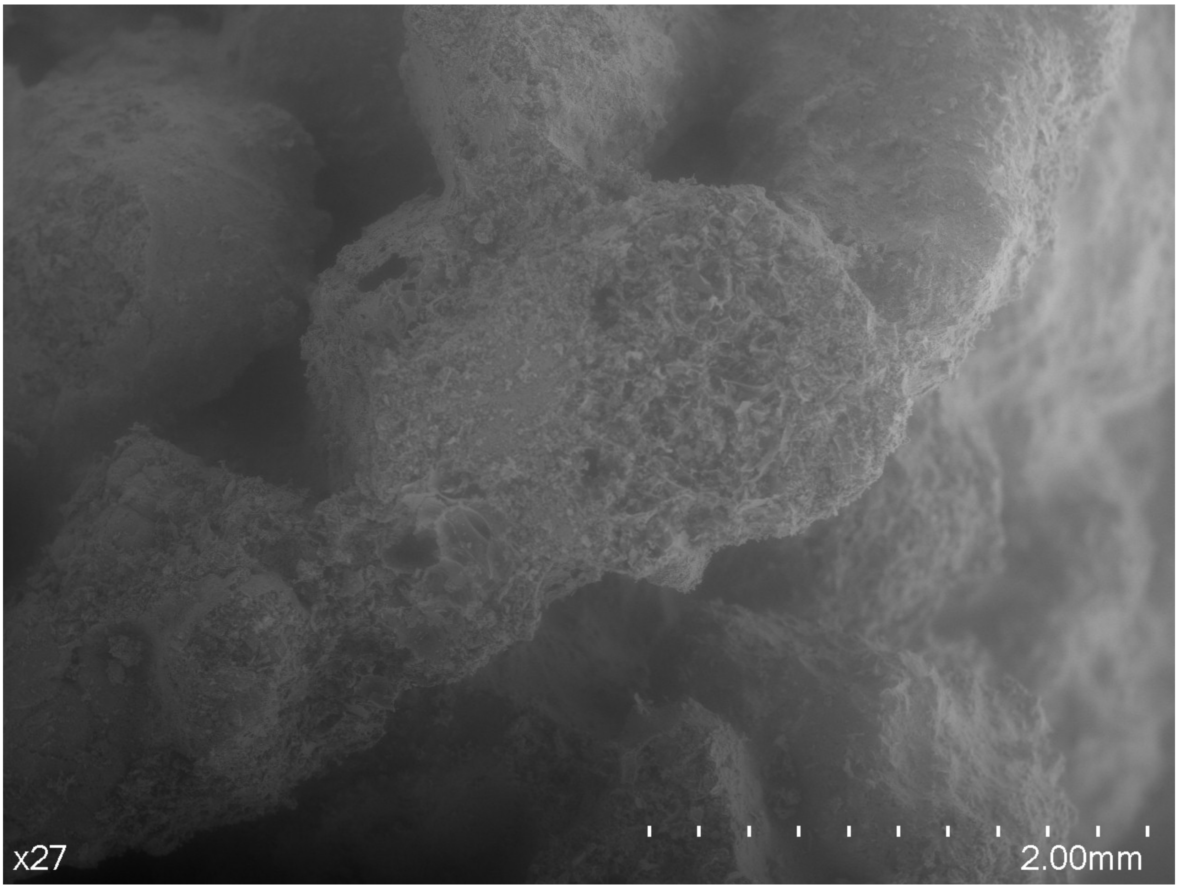

Figure S22. M-SG1—SEM image of expanded perlite and cement interface at M-SG.

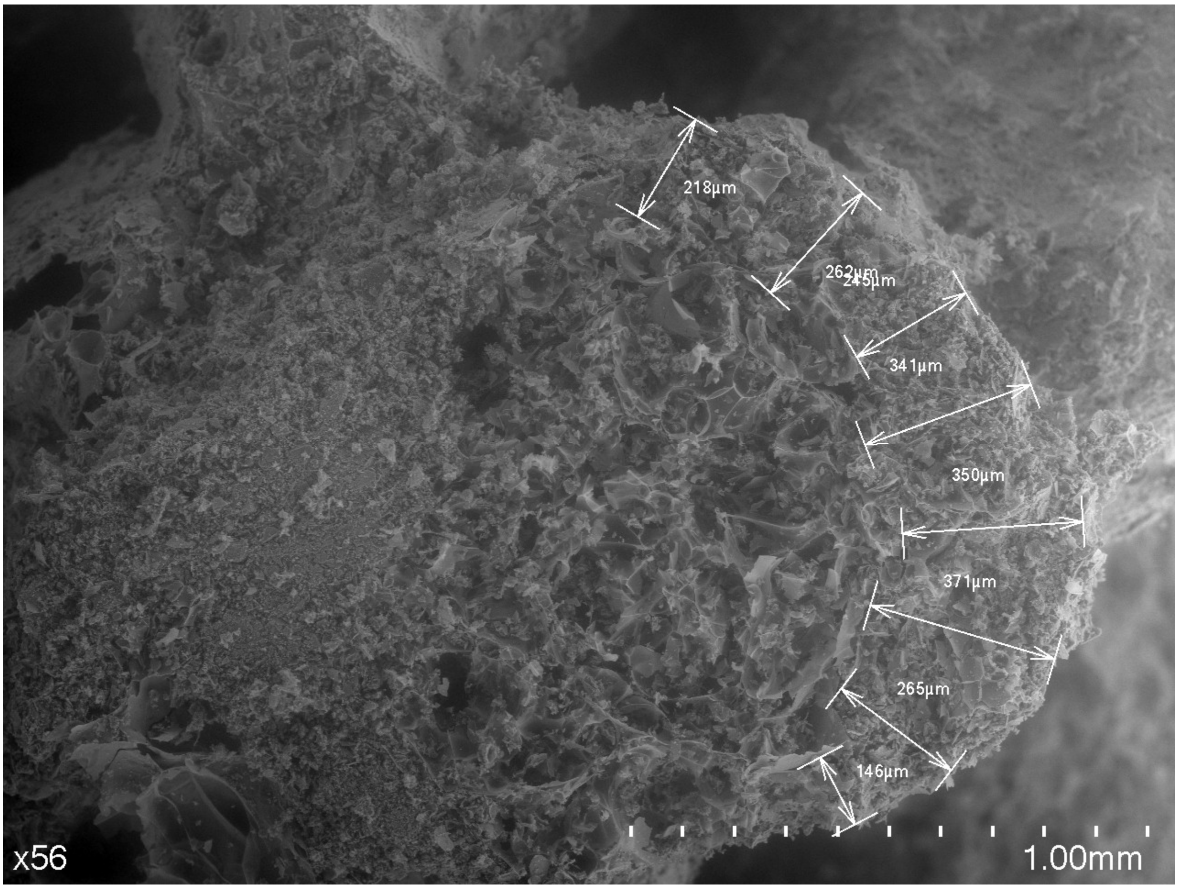

Figure S23. M-SG2—SEM image of expanded perlite and cement interface at M-SG.

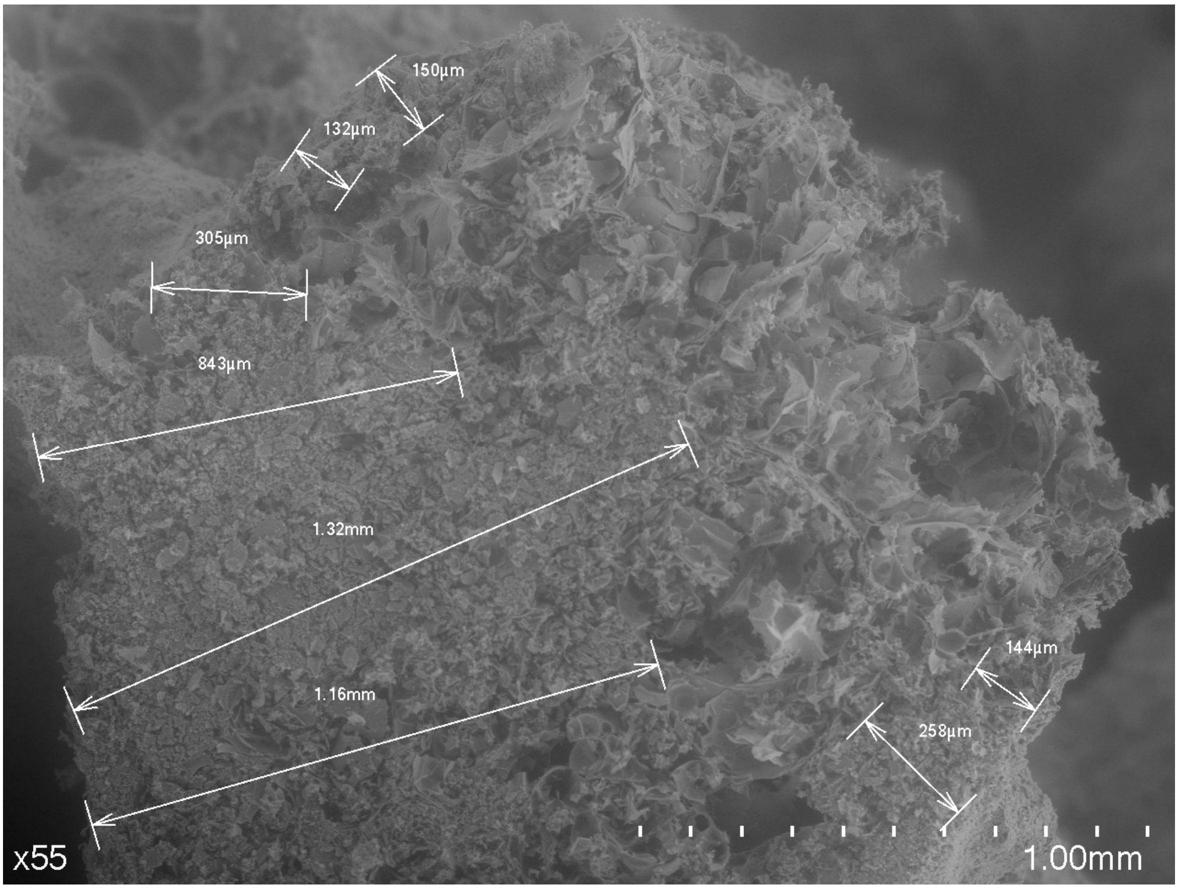

Figure S24. M-SG3—SEM image of expanded perlite and cement interface at M-SG.

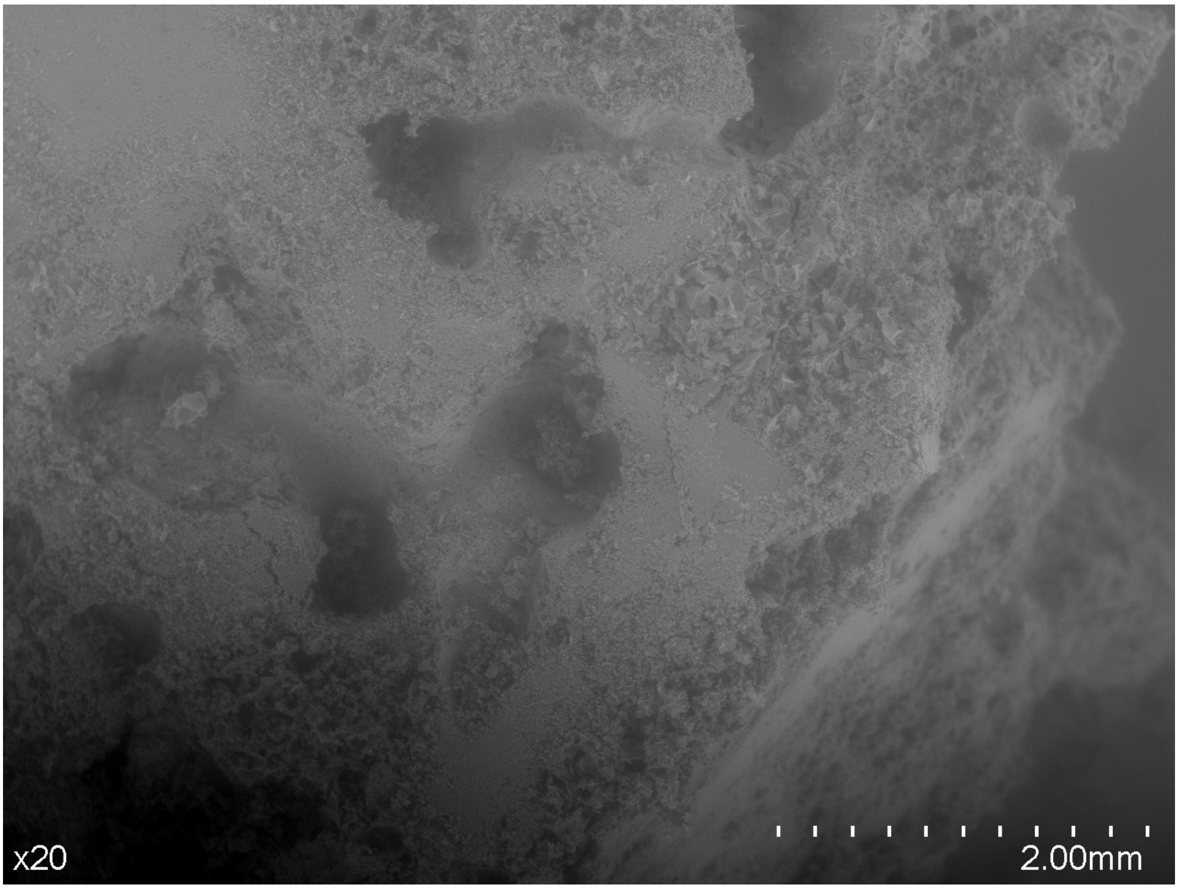

Figure S25. F-SG1—SEM image of expanded perlite and cement interface at F-SG.

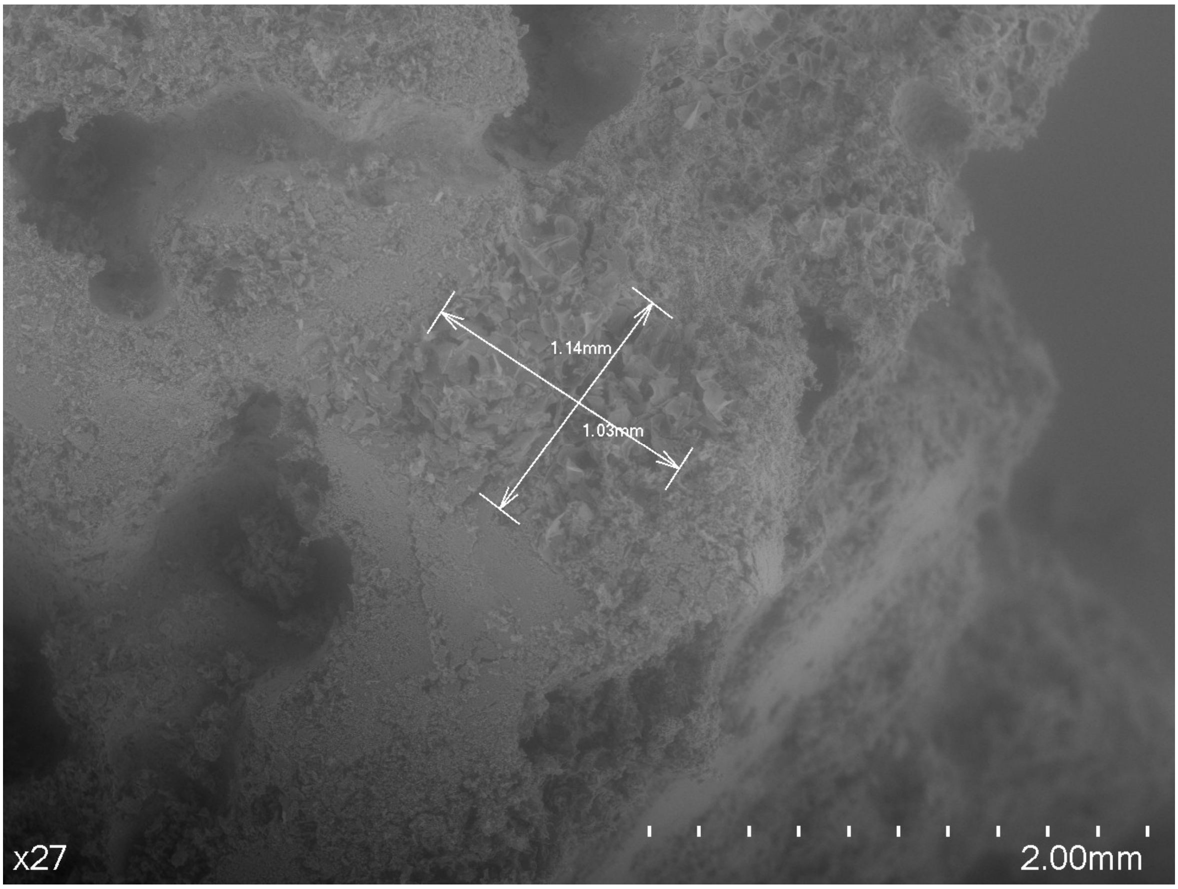

Figure S26. F-SG2—SEM image of expanded perlite and cement interface at F-SG.

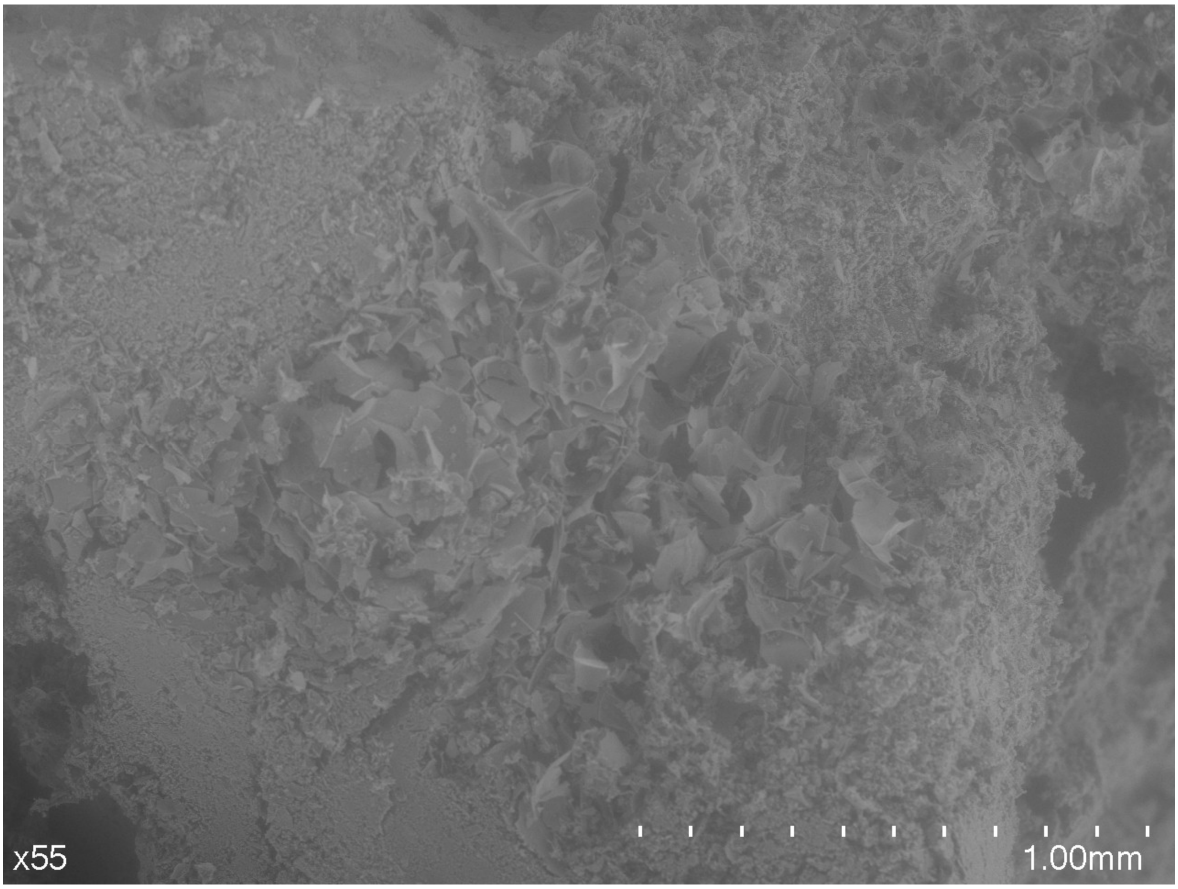

Figure S27. F-SG3—SEM image of expanded perlite and cement interface at F-SG.
